# Application of Electrospun Drug-Loaded Nanofibers in Cancer Therapy

**DOI:** 10.3390/polym16040504

**Published:** 2024-02-12

**Authors:** Yaoyao Yang, Rui Zhang, Zhiyuan Liang, Junli Guo, Bingying Chen, Shengwei Zhou, Dengguang Yu

**Affiliations:** School of Materials and Chemistry, University of Shanghai for Science and Technology, 516 Jungong Road, Shanghai 200093, China; 212203081@st.usst.edu.cn (R.Z.); 212203084@st.usst.edu.cn (Z.L.); 223353223@st.usst.edu.cn (J.G.); 223353224@st.usst.edu.cn (B.C.); 213353182@st.usst.edu.cn (S.Z.)

**Keywords:** electrospinning, nanofibers, nanostructure, drug release, cancer therapy

## Abstract

In the 21st century, chemotherapy stands as a primary treatment method for prevalent diseases, yet drug resistance remains a pressing challenge. Utilizing electrospinning to support chemotherapy drugs offers sustained and controlled release methods in contrast to oral and implantable drug delivery modes, which enable localized treatment of distinct tumor types. Moreover, the core–sheath structure in electrospinning bears advantages in dual-drug loading: the core and sheath layers can carry different drugs, facilitating collaborative treatment to counter chemotherapy drug resistance. This approach minimizes patient discomfort associated with multiple-drug administration. Electrospun fibers not only transport drugs but can also integrate metal particles and targeted compounds, enabling combinations of chemotherapy with magnetic and heat therapies for comprehensive cancer treatment. This review delves into electrospinning preparation techniques and drug delivery methods tailored to various cancers, foreseeing their promising roles in cancer treatment.

## 1. Introduction

The shape and location of tumors vary, typically categorized as benign or malignant. Benign tumors usually cause minimal harm, often resulting in localized obstructions and pressure. Malignant tumors, known as “cancer”, consist of cells differing from normal ones in the body. While normal cell growth is limited, cancer cells proliferate without restraint. As they multiply, cancer cells consume essential nutrients meant for normal cells, leading to the decline and demise of healthy cells. Additionally, these cancer cells produce toxins that harm the body. Chemotherapy is a vital aspect of cancer treatment, aiming to impede the division and growth of cancer cells. However, chemotherapy also brings about systemic side effects due to its high toxicity, drug resistance, and limited selectivity [1]. Challenges in tumor therapy include rapid drug elimination, insufficient delivery to tumor tissues, and the risk of metastasis to other organs. Consequently, cancer chemotherapy often results in dose-related toxicity and side effects such as immunosuppression, neuropathy, and hair loss [2].

**Figure 1:**
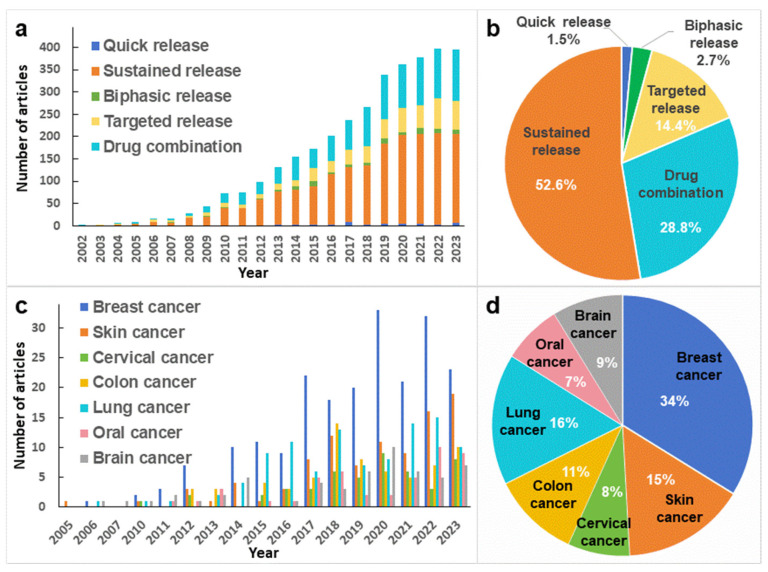
Statistics of studies retrieved from the literature on the “Web of Science” platform with the subjects of (**a**,**b**) “electrospinning & drug delivery” and (**c**,**d**) “electrospinning & cancer”, respectively.

Electrospinning can be used to incorporate drugs into nanofibers, resulting in a decelerated drug release rate, extended release duration, minimized drug dispersal, and effective mitigation of potential adverse effects from multiple-drug delivery [3]. Moreover, electrospun drug-loaded nanofibers have the capacity to concurrently accommodate various drugs, fostering a synergistic effect among them. This synergy revitalizes initially drug-resistant compounds, generating novel therapeutic effects [4,5]. Figure 1 depicts the upward trajectory of articles published in the past 20 years on the “Web of Science” database, focusing on “electrospinning & drug delivery” and “electrospinning & cancer”, respectively. As shown in Figure 1a,b, the data reveal that the applications of electrospinning in drug delivery continues to increase, with the primary research areas including sustained release, drug combination, and targeted release. The data in Figure 1c,d demonstrate the growing application of electrospinning in cancer treatment research. Most of the studies are about lung cancer and breast cancer, and research on other types of cancer is also increasing year by year.

The primary aim of this review is to assess the efficacy and role of electrospun nanofibers in addressing the formidable challenges of chemotherapy in cancer treatment, emphasizing their potential in mitigating drug-related toxicity and enhancing drug delivery efficiency.

## 2. Preparation of Drug-loaded Nanofibers via Electrospinning

**Figure 2:**
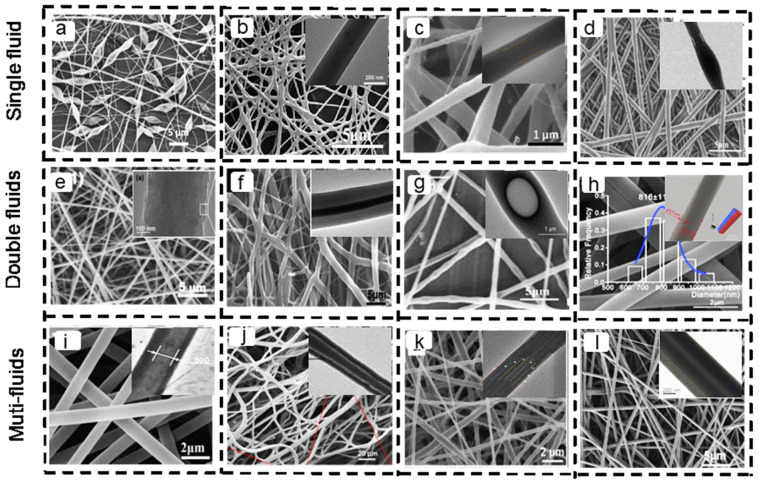
SEM and TEM images of electrospun drug-loaded nanofibers with various structures. Single-fluid electrospinning: (**a**) Bead-on-string fibers [7]. Copyright 2018, reproduced with permission from *Nature*. (**b**) Smooth fibers [8]. Copyright 2022, reproduced with permission from MDPI. (**c**,**d**) Core–sheath fibers [10,11]. Copyright 2023, 2019, reproduced with permission from Elsevier. Double-fluid electrospinning: (**e**) Smooth fibers [9]. Copyright 2021, reproduced with permission from MDPI. (**f**) Core–sheath fibers [12]. Copyright 2020, reproduced with permission from Elsrvier. (**g**) Beaded core–sheath fibers [13]. Copyright 2019, reproduced with permission from WILEY. (**h**) Janus fibers [17]. Copyright 2021, reproduced with permission from Elsevier. Multi-fluid electrospinning: (**i**) Two-layer core–sheath fibers [14]. Copyright 2016, reproduced with permission from Elsevier. (**j**) Three-layer core–sheath fibers [15]. Copyright 2020, reproduced with permission from MDPI. (**k**) Four-layer core–sheath fibers [16]. Copyright 2021, reproduced with permission from Elsevier. (**l**) Two core fibers and one sheath fiber [18]. Copyright 2020, reproduced with permission from Elsevier.

Electrospinning is an electrohydrodynamic technique used to create nanofibers. This process involves energizing polymer droplets to generate jets. The electrostatic repulsion between the charges generated on the droplets causes them to form a Taylor cone, which elongates due to stretching induced by the electric current, resulting in the production of fine fibers. Substances that are suitable for electrospinning are primarily organic polymers present in a solution or in a molten state. These include small-molecule polymers that dissolve in conductive polymers at optimal dissolution concentrations or those that can self-assemble to achieve sufficient chain entanglement [6]. Currently, as shown in Figure 2, advancements in multi-fluid electrospinning technology have enriched the structures of electrospinning fibers. Varieties such as bead-on-string structures [7], smooth fibers [8,9], core–sheath structures [10,11,12,13,14,15,16], Janus structures [17], “pig nose” structures [18], and others are continually emerging.

### 2.1. Single-Fluid Electrospinning

Single-fluid electrospinning stands as a commonly used technique to produce nanofibers. The morphology of these electrospun fibers is influenced by system parameters (polymer molecular weight, solvent properties, spinning liquid concentration, etc.), process factors (voltage, flow rate, and acceptance distance), and environmental parameters (temperature and humidity).

Typically, smooth and continuous nanofibers are the most prevalent morphologies. Noteworthy patterns include a decreasing solution viscosity, leading to a smaller fiber diameter or increased fusiform defects, and a higher average polymer molecular weight favoring easier bonding and network formation [19]. A disruption in the balance between the surface tension and electric field force can result in jet splitting and fiber branching [20]. Common substrates for drug-carrying nanofibers include polyvinylpyrrolidone (PVP) [7], polycaprolactone (PCL) [21,22], and cellulose acetate (CA) [23]. Loading drugs onto these nanofibers enables the rapid, controlled, and targeted release of the drug. Using various polymers to create composite fibers for drug loading not only ensures effective drug release properties but also enhances the overall fiber properties [24,25].

When preparing drug-loaded nanofibers using electrospinning, it is crucial to consider the compatibility between the drugs and polymer substrates. For example, if hydrophilic drugs are paired with hydrophobic substrates, this can cause drug aggregation within the fibers, resulting in uneven drug dispersion [26].

Post-processing, drugs with low water solubility can coexist with the drug carriers in two ways: homogeneous recombination, where drug molecules are evenly distributed among the drug carrier molecules, and heterohybridization, where pure drug nuclei are enveloped in polymer shells to create drug nanorepositories [27].

Single-fluid electrospinning can create beaded structures, such as continuous spherical or spindle structures connected to filaments. Bead formation occurs on bead-on-string fibers when the dilute polymer solution’s jet breaks, creating oval protrusions along the fibers. Additionally, stretching low-viscosity polymers leads to particle formation through the entanglement of a single polymer chain at the molecular level [28]. Achieving high feed rates and spinning voltages is crucial for bead formation in polymer production. Factors such as surface tension and polymer concentration significantly influence this process. Introducing physical crosslinking agents or salts can enhance the electrical spinning capacity of the polymer solution, facilitating bead structure formation [29].

Initially seen as a by-product of smooth fibers, recent research has highlighted the distinct advantages of bead structures. These structures possess a higher specific surface area compared to single uniform fibers, offering unique benefits in drug loading [30], sustained release [31], and tissue engineering [32]. Studies indicate that controlling the bead and wire diameters affects the drug embedding depth and loading rate. Precisely managing the bead diameter enables the efficient encapsulation of micron-sized drug particles [33]. Furthermore, in conjunction with the separation of water and oil through emulsion electrospinning, single-fluid electrospinning can generate nanofibers with a core–sheath structure [34].

### 2.2. Double-Fluid Electrospinning

Coaxial electrospinning and the widely used double-fluid electrospinning process are frequently employed to create nanofibers with core–sheath structures. Removing the core layer of these electrospun core–sheath nanofibers via calcination or washing yields hollow nanofibers [35,36]. Moreover, by electrospinning two working fluids through eccentric spinning heads, drug-loaded nanofibers with parallel [37] or bead [38] structures can be produced. Investigating the connection between fiber structures and drug-release properties while utilizing double-fluid electrospinning technology to craft various types of drug-carrying nanofibers with distinct structures is a prominent focus in controlled drug-release research.

Creating core–sheath nanofibers through coaxial electrospinning is relatively straightforward and offers significant control. However, managing variables in this process is more intricate compared to single-fluid electrospinning. Alongside controlling factors such as the power supply voltage, collection distance, and flow rates for the core and sheath layers, it is also essential to consider the compatibility of the solutions used in these layers. By regulating the polymer matrix, the type and spatial arrangement of drug storage, and the solubility of the loaded drug, various objectives such as rapid release [39], sustained release [40,41], pH-responsive release [12], and multidrug combination [42] can be effectively achieved, paving the way for the development of advanced oral Drug Delivery Systems. For example, controlling the sheath thickness in the core–sheath structure can be accomplished by adjusting the flow rate of the sheath fluid and the polymer concentration, thereby serving as a crucial promise to modify the drug release profile [8]. Furthermore, introducing pore-forming agents to the sheath enables adjustments in the fiber’s hydrophilicity, allowing for the precise manipulation of the sheath surface properties and consequently refining the drug release patterns [10,43].

Earlier studies suggested that both the core layer and sheath working fluid in coaxial electrospinning needed to be electrospinnable for the successful preparation of a core–sheath nanostructure [44]. However, Yu et al. developed an enhanced coaxial electrospinning method that employs a non-spinnable liquid as the sheath working fluid, challenging this belief [45]. This breakthrough enabled the creation of novel drug-loaded sheath nanofibers, which exhibited delayed quick release, prolonged slow-release, reduced tail break release, and the complete release of drug molecules. Non-spinnable liquids encompass pure solvents, surfactants, or salt solutions, significantly broadening the application scope of electrospun nanofibers [46,47].

### 2.3. Multi-Fluid Electrospinning

In multi-fluid electrospinning, at least one spinning fluid (the main fluid) must be spinnable and take the lead role, whereas other fluids can be non-spinnable. Regardless of the number of fluids, the fundamental principle is to maintain only one compound as the Taylor cone, a single direct flow, and a solitary unstable region. These principles govern multi-fluid electrospinning [48].

When fluids run in parallel, the repulsion of similar charges can cause fluid splitting under electric fields, disturbing the creation of Janus nanostructures [49] and posing a challenge to multi-fluid electrospinning. The high polymer concentration in shell solutions promotes encapsulation but also increases the risk of frequent blockages. Shell solutions with low polymer concentrations seldom cause blockages, whereas the core solution penetrating the sheath solution results in encapsulation failure [50]. Several factors contribute to the increased complexity of multi-fluid electrospinning compared to both single-fluid and double-fluid electrospinning processes.

The advancement of multistage structured electrospun nanofibers holds significant promise in biomedical applications. These fibers enable the release of two or more drugs at varying rates from the membrane [15,51], or they can modulate the release of a single drug at different rates, such as by incorporating distinct drugs in each layer of three-layered core–sheath nanofibers. By adjusting the drug content and release rates based on layer thickness, a remarkable 94.2% maximum killing rate of MCF-7 breast cancer cells has been achieved [52].

In modified triaxial electrospinning, increasing the intermediate fluid flow leads to an increased sheath thickness. Thicker-sheathed core–sheath fibers displayed an almost linear zero-order release pattern [53].

In short, multi-fluid electrospinning, exemplified by triaxial electrospinning, allows for precise control over the layer thickness or drug-loaded fiber attributes. This control is achieved by manipulating the flow type, flow rate ratio, or fluid concentrations of different working fluids, resulting in tailored, specific drug release performance [54,55,56,57].

## 3. Drug Release Modes of Drug-Loaded Nanofibers

The drug release mechanisms of drug-loaded nanofibers encompass rapid release, continuous release, multistage release, biphasic release, and delayed release, each contingent upon the drug’s release rate. These diverse release types offer distinct advantages in therapeutic applications. Rapid release finds primary utility in addressing pain, fever, and heart diseases [58]. Continuous release maintains a steady blood drug concentration, making it better suited for chronic diseases [14,59,60,61].

**Figure 3:**
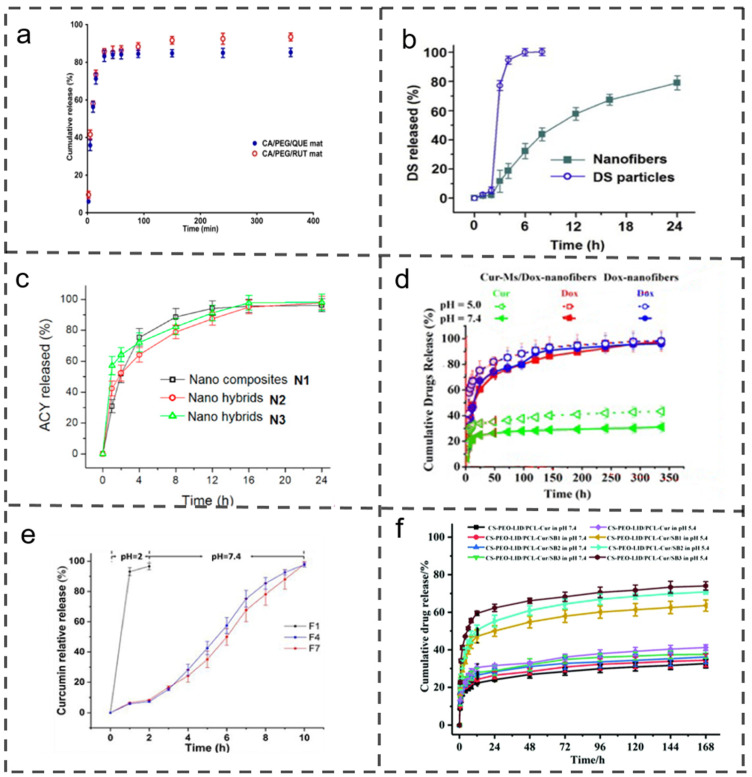
Drug release curves. (**a**) Quick release [58]. Copyright 2022, reproduced with permission from MDPI. (**b**) Sustained release [14]. Copyright 2016, reproduced with permission from Elsevier. (**c**) Biphasic release [9]. Copyright 2021, reproduced with permission from MDPI. (**d**) Dual drug release [63]. Copyright 2014, reproduced with permission from WILEY. (**e**) pH response [64]. Copyright 2023, reproduced with permission from MDPI. (**f**) pH sensitivity [65]. Copyright 2020, reproduced with permission from RSC.

Biphasic release not only rapidly alleviates symptoms but also sustains a consistent drug concentration in the bloodstream, thereby enhancing efficacy in patients [62]. Additionally, targeted release and drug combinations represent more specialized release modes. Figure 3 provides an overview of the release curves corresponding to these different release modes in drug-loaded electrospun nanofibers [9,63,64,65]. Table 1 summarizes the typical drug-release methods and indications for electrospun drug-loaded nanofibers [9,14,25,58,61,64,66,67,68,69,70,71,72,73].

### 3.1. Quick Release

In Figure 3a, hydrophilic polymer substrates, such as PVP and polyethylene glycol (PEG), promote the rapid release of drugs from drug-loaded electrospun nanofibers. Stoyanova et al. successfully prepared new fiber materials containing natural biologically active compounds like quercetin (QUE) and rutin (RUT) using cellulose derivatives such as CA, along with water-soluble polyether and PEG as raw materials. The integration of polyether into the developed fiber material enhanced the membrane’s hydrophilicity and wettability, accelerating the in vitro release of bioactive compounds. Within 360 min, approximately 93.5% and 85.3% of RUT and QUE, respectively, were released from the fiber membrane, demonstrating a potent cytotoxic effect on cancer cells. Notably, the cytotoxic effect on non-cancer cell lines decreased by 3 to 4.5 times, indicating potential applications in localized cervical tumor treatments and wound dressing [58].

In another study, Bukhary et al. utilized polyvinylpyrrolidone as a polymer matrix, employing electrospinning to create fast-dissolving oral Fixed-Dose Combinations (FDCs) containing amlodipine besylate and valsartan, which are commonly used drugs for hypertension. These FDCs exhibited drug encapsulation rates exceeding 85% of the theoretical dose. Achieving a rapid release within 360 s, the resulting fiber membrane demonstrated both folding durability and adequate thickness, rendering it suitable for use as an oral patch [67].

### 3.2. Sustained Release

Sustained release stands out as a protracted mode of drug delivery that is extensively employed in treating chronic conditions like cancer. For instance, biodegradable electrospun scaffolds, composed of chitosan (CS), polyvinyl alcohol, ibuprofen, and silver nanoparticles, exhibit both analgesic and antibacterial effects, and they are applied in tissue engineering [74].

In another application, electrospun nanofibers, supported by the hydrophobic polymer poly(lactic-co-glycolic acid) copolymer (PLGA) with pirfenidone as the outer layer and the hydrophilic polymer PVP with moxifloxacin as the inner layer, extend the release duration of both drugs. This extended release significantly prolongs drug residence on corneal abrasions, reducing the frequency of doses [75].

Resveratrol (RES) combined with other natural drugs exhibits inhibitory effects on multiple pathways, influencing cancer cell growth and cancer-causing signaling. In RES- and xanthohumol (XAN)-loaded fiber membranes, produced via coaxial electrospinning, drug release accelerates with an increased drug content. RES and XAN are released progressively, and their releases are sustained for up to 350 h [76].

Effective local chemotherapy drug delivery to tumors necessitates the development of Drug Delivery Systems (DDSs), ensuring long-term drug release. Several DDSs for local drug delivery, including polymer nanoparticles, hydrogels, nanoemulsions, and nanofibers, have been developed [77]. Electrospun drug-loaded nanofibers, functioning as carriers for amorphous drugs, enhance drug bioavailability compared to conventional drugs. This characteristic minimizes the total dosage and administration frequency, offering an advantage in cancer therapy.

### 3.3. Biphasic Release

Biphasic release offers the combined advantages of rapid and sustained release, holding promising applications in various fields such as cancer treatment [78,79,80], bone tissue regeneration [81,82,83,84], wound healing [85,86,87,88], rotator cuff tendon tears [89], and eye diseases [90].

When preparing electrospun drug-loaded nanofibers, due to the rapid evaporation of solvents and high ionic strength in the solution, most drug compounds tend to accumulate on or near the fiber surface, leading to an initial burst release. This initial burst release is unavoidable [91]. Consequently, the biphasic release rate of drug-loaded nanofibers is influenced by factors such as the polymer molecular weight [92], concentration [93], sheath thickness [94], additives, and more. For instance, introducing graphene oxide can enhance the mechanical properties of electrospun membranes and slow the release of model drugs by reducing the matrix swelling [95].

Apart from the nanofiber composition, the internal structures and external shapes of the nanofibers play significant roles in determining the drug release rate [17].

### 3.4. Targeted Release

Variations in pH across different parts of the human body inspire the use of pH-sensitive materials, like the Eudragit polymer family, for targeted drug delivery [96,97]. For instance, the gastric juice pH ranges from 0.9 to 1.8, contrasting with the intestinal tract’s pH of around 7. Electrospun core–sheath nanofibers, composed of a lipid drug core and an Eudragit S100 shell, exhibit promise in providing targeted drug release, specifically to the colon [14].

The slightly elevated acidity of the tumor microenvironment (TME) compared to normal physiological conditions presents an opportunity for pH-responsive DDSs in tumor therapy [98]. An example is γ-cyclodextrin (γ-CD) linked with 3-(diethylamino)propylamine (DEAP), electrospun to create microparticles with a cellular porous structure for encapsulating paclitaxel (PTX). Under acidic conditions, the protonation of pH-responsive DEAP disrupts the particle structure via charge repulsion, triggering the release of PTX from the collapsed particle [99]. In another approach, core–sheath nanofibers, prepared via coaxial electrospinning, feature chitosan and polyethylene oxide as the sheath, and polycaprolactone with sodium bicarbonate as the core. The sheath and core layers are loaded with lidocaine hydrochloride (Lid) and curcumin (CUR), respectively. The acidic environment protonates the –NH_2_ group of chitosan, forming –NH_3_^+^, whereas sodium bicarbonate reacts with hydrogen ions to generate CO_2_, creating pores on the fiber surface. Consequently, both Lid and CUR display release curves that are responsive to an acidic pH (~5.4). Due to chitosan’s sensitivity to acidity, the total release of Lid was higher under acidic conditions than under alkaline conditions [66].

### 3.5. Drug Combination

Drug resistance easily develops during tumor treatment, often necessitating the use of two or more antitumor drugs in combination. This approach is applicable to cancers that have failed chemotherapy and targeted therapy or exhibit frequent drug resistance. Such scenarios are commonly observed in the treatment of glioblastoma [100], triple-negative breast cancer [101], chronic myeloid leukemia [102], and other cancers [103].

**Figure 4:**
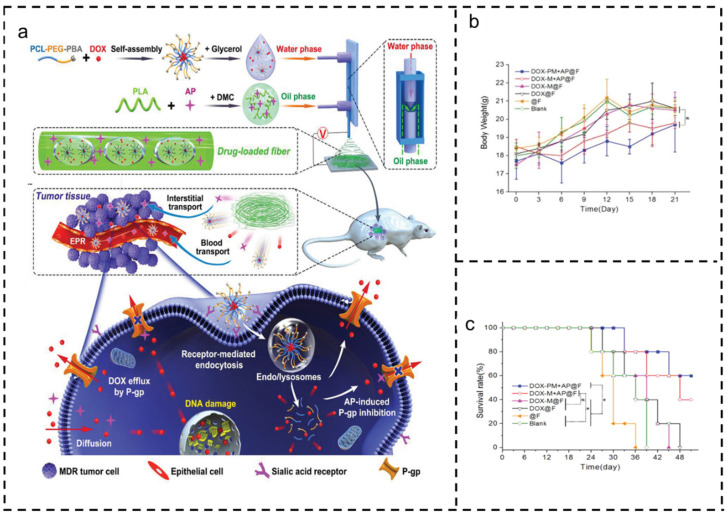
Application of doxorubicin and apatinib in combination to overcome multidrug resistance in cancer [13]. Copyright 2019, reproduced with permission from WILEY. (**a**) Fabrication and application diagram of drug-loaded fiber device. (**b**) Weight change. (**c**) Survival curves of tumor-bearing nude mice.

The natural drug PTX stands as one of the most commonly used drugs in combination therapies. It demonstrates synergic effects with evolimus [100], tetrandrine [104], CUR [105], silybin (SB) [106], melittin [107], honokiol (HNK) [108], fatty acid amide hydrolase (FAAH) [109], and epirubicin (EPI) [110]. The combination of PTX and HNK enhances the cytotoxicity of PTX in drug-resistant MCF-7/ADR tumor cells by increasing paclitaxel’s cellular uptake and preventing the invasion and migration of MDA-MB-231 cells, both in vitro and in vivo, thereby reducing metastasis in nude mice [108]. Electrospun fibers loaded with paclitaxel and tetrandrine inhibit the expressions of p-STAT3 and p-JAK, initiate the release of cytochrome c, and promote the expression of caspase protein [104]. Moreover, the combined treatment of PTX and silybin (SB) exhibits a synergistic effect by sensitizing chemotherapy and regulating the tumor microenvironment, effectively inhibiting tumor growth through enhanced intratumoral penetration [106]. Moreover, doxorubicin (DOX) exhibits synergistic effects with berberine (BBR) [111], CUR combined with resveratrol [112], and apatinib (AP) [13]. Y. He et al. demonstrated the simultaneous loading of DOX and AP onto nanofibers using microfluidic electrospinning, as depicted in Figure 4. Achieving an encapsulation efficiency of 99%, the two drugs followed a preset release model: the rapid release of DOX and the gradual release of AP. Sustained AP release effectively inhibits drug resistance in tumor cells, while the increased intracellular accumulation of DOX leads to outstanding antitumor results with minimal systemic toxicity [17].

## 4. Application of Drug-loaded Nanofibers in Cancer Therapy

As previously highlighted, electrospun drug-loaded nanofibers offer high drug loading, exceptional encapsulation efficiency, and specific drug release profiles, including sustained, multiphase, targeted, and combined release. These capabilities are achieved through meticulous material selection and design, presenting specific and extensive applications in cancer treatment [113,114,115]. This section delineates their applications across various tumor therapies.

### 4.1. Breast Cancer

Breast cancer stands as one of the most prevalent cancers in today’s society, and it is commonly treated with surgical resection or post-tumor-resection chemotherapy. Electrospun fibers serve as vehicles for chemotherapy drug delivery localized to the site [116], enabling dosage adjustment based on patch thickness and total area [117]. Abasalta et al. utilized coaxial electrospinning to load DOX, an anticarcinostatic drug, onto nanofibers. The presence of DOX-loaded coaxial nanofibers resulted in a mortality rate of approximately 57% for MCF-7 breast cancer cells after 7 days [118].

Another development by Li et al. introduced an implantable hierarchical microfiber device using coaxial electrospinning, forming periodically arranged chambers within a fiber matrix. Doxorubicin hydrochloride within these chambers exhibited rapid release, effectively eliminating the remaining tumor cells and curbing tumor recurrence. Additionally, the continuous release of the matrix metalloproteinases-2 (MMP-2) inhibitor disulfide from the fiber matrix inhibited tumor invasion and prevented metastasis. This fiber-based device showed promising results as a postoperative local adjuvant chemotherapy, demonstrating excellent efficacy in preventing recurrence and metastasis [119]. Studies have demonstrated that the addition of surfactants to electrospun fibers improves their therapeutic effects [120,121].

Enhancing chemotherapy’s efficacy often involves combining it with complementary cancer treatment methods like magnetic therapy [122,123], radiation therapy [124], and hyperthermia [125], among others. Hence, the integration methods of metal or magnetic nanoparticles with electrospinning are common approaches. For instance, spherical nickel ferrite nanoparticles synthesized via the sol-gel method were employed in electrospinning to produce magnetic core–sheath nanofibers loaded with DOX, achieving an encapsulation efficiency exceeding 90%. These drug-loaded nanofibers exhibited sustained and controlled DOX release for 25 days under physiological pH conditions and within 7 days under acidic pH conditions without an initial burst release. In the presence of an external magnetic field, these nanofibers demonstrated up to 83% maximum cytotoxicity toward MCF-7 breast cancer cells, showcasing their potential to be used for breast cancer treatment [126].

Another example involved incorporating the anticancer drug CUR into mesoporous silica nanoparticles (MSNs) embedded in PLGA via electrospinning. This approach examined CUR’s in vitro release mode from the fibers and its anticancer efficacy against MCF-7 breast cancer cells. The findings revealed sustained and prolonged drug release properties for CUR, with the drug-loaded fibers exhibiting higher cytotoxicity and reduced mobility, thereby increasing apoptosis induction in cancer cells [127].

**Figure 5:**
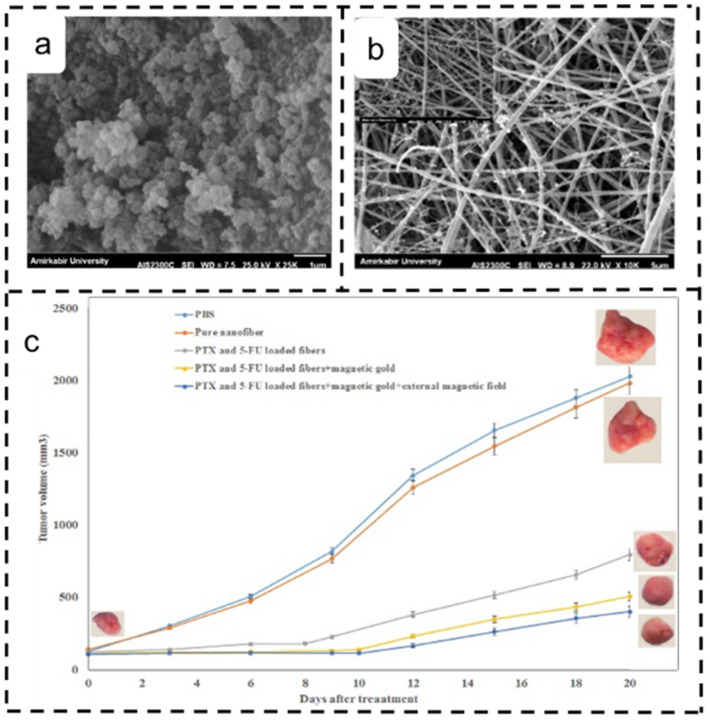
Application of electrospun magnetic core–sheath drug-carrying nanofibers in breast cancer treatment [128]. Copyright 2020, reproduced with permission from Elsevier. SEM images of (**a**) magnetic gold nanoparticles and (**b**) nanofibers coated with magnetic gold nanoparticles. (**c**) Tumor growth after fibroblast treatment.

Moreover, in a setup where the anticancer drugs paclitaxel and 5-Fluorouracil (5-FU) were incorporated into the core layer of chitosan-containing core–sheath nanofibers, magnetic gold nanoparticles coated the nanofibers’ surfaces. The high swelling of the chitosan copolymer and nanofibers led to an initial rapid release of paclitaxel and 5-FU within 48 h, followed by a sustained release for up to 25 days. This super-strong and sustained release profile of paclitaxel and 5-FU, facilitated by the gold nanoparticle layer, resulted in a 78% maximum killing rate of magnetic gold-coated nanofibers on 4T1 breast cancer cells when an applied magnetic field was present [128] (Figure 5).

### 4.2. Skin Cancer

Skin cancer manifests primarily in three forms: basal cell carcinoma, squamous cell carcinoma, and the highly malignant melanoma, which is particularly prone to local invasion and distant metastasis. Surgery serves as the primary treatment for most melanomas. However, owing to their invasive nature, residual tumor cells post-surgery significantly elevate the risk of local recurrence and micrometastase. Consequently, drug-loaded electrospun fiber membranes are frequently applied in postoperative chemotherapy for melanoma, commonly utilized as patches [129,130] or implantable devices [131,132].

S. Samadzadeh et al. developed temperature-responsive single-layer electrospun nanofibers with dual capabilities of simultaneous heating and two-stage drug release, triggered by an alternating magnetic field switch. Their smart hyperthermia nanofiber scaffolds exhibited a combination of rapid initial drug release followed by prolonged release. Through the dual effects of heat and the two-stage drug release, they effectively induced cytotoxicity, harnessing synergistic effects over an extended period. This two-stage drug release method, compared to standard chemotherapy, offers preferable cytotoxicity induction through prolonged synergistic effects. These temperature-responsive drug-loaded electrospun nanofibers could serve as effective implantable devices to prevent local cancer recurrence [132].

Muthulakshmi et al. extracted bioactive compounds from the medicinal plant Terminalia catappa (TC), loading them into a sodium alginate polymer. Electrospun fiber membranes prepared from this combination exhibited the ability to heighten oxidative stress and induce apoptosis by regulating caspase protein in skin melanoma cells, showcasing remarkable efficacy. The presence of anticancer elements within TC contributes to its enhanced anticancer activity, while the electrospinning technique plays a crucial role in amplifying this effect [133].

**Figure 6:**
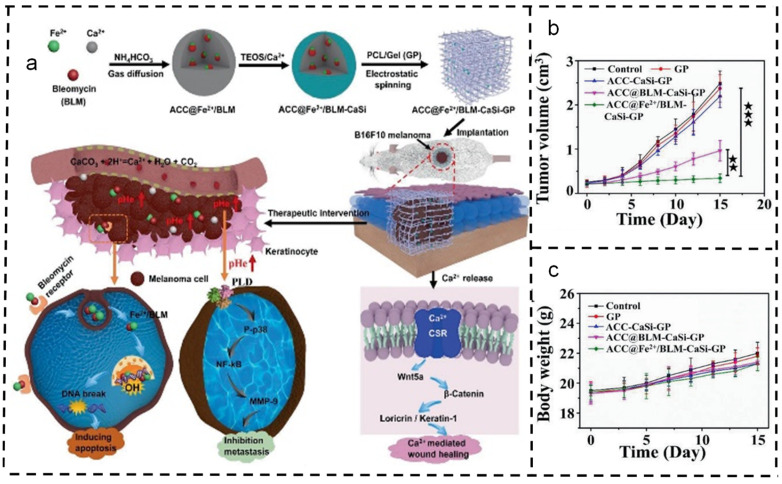
Application of gelatin/polycaprolactone (GP) nanofiber scaffolds in inhibiting proliferation of skin cancer cells [134]. Copyright 2021, reproduced with permission from WILEY. (**a**) The synthesis process of the ACC@Fe^2+^/BLM-CaSi-GP stent and its complementary ability to simultaneously treat melanoma and wound healing. (**b**) Weight change (n = 5) and (**c**) survival curves of nude mice bearing tumors under different treatments with MCF-7/ADR at 21 days.

In another approach, Xue et al. engineered a therapeutic amorphous calcium carbonate (ACC) nano-formulation within a single layer of gelatin/polycaprolactone (GP) nanofibers using electrospinning. Combining ACC nano-formulation with Fe^2+^-pre-activated bleomycin results in a biocatalytically enhanced therapeutic effect. Hydrolyzed ACC functions as a proton scavenger, effectively adjusting the acidity of tumor tissues in situ, thereby continuously suppressing tumor recurrence and metastasis. The acid-triggered decomposition of ACC releases Ca^2+^, activating the downstream Wnt/β-catenin signaling pathway. This process, combined with the wound regeneration effect of GP substrates, provides adjunct therapy for the postoperative management of melanoma [134] (Figure 6).

Combining chemotherapy, magnetic therapy, and hyperthermia represents a significant trend in cancer treatment. Cimen et al. utilized electrospinning to fabricate spherical silver nanoparticles (AgNPs) coated with thyme extract using the bioreduction method, embedding them in a polycaprolactone/polylactic acid (PCL/PLA) core–sheath nanofiber scaffold. SiO_2_ and Ag nanoparticles were integrated into the polymer during preparation. This method facilitates the actual treatment combination of hyperthermia and magnetic therapy while exhibiting substantial antibacterial effects against Staphylococcus aureus and Escherichia coli. Demonstrating a higher efficiency compared to other nanofiber scaffolds, it notably inhibits the proliferation of skin cancer cells (SK-MEL-30 cells). Additionally, the fiber membrane exhibits good biodegradability, mitigating harm to patients after repeated removal [135].

### 4.3. Cervical Cancer

Vaginal drug administration offers several advantages, including ease of application, prolonged drug retention, and extensive coverage, and it is commonly employed in cervical cancer treatment. However, traditional carriers like gels and tablets face limitations such as instability, leakage, mucus adhesion, and an inadequate residence time. Consequently, drug-carrying fiber membranes for cervical cancer treatment often require enhanced adhesion. Aggarwal et al. investigated polycaprolactin/CS loaded with cisplatin composite electrospun nanofibers, demonstrating superior mucosal adhesion and exceptional efficacy in local chemotherapy for cervical cancer in mice [136]. Additionally, Yi et al. and B. Chen et al. developed biodegradable drug-loaded nanofibers capable of sustained drug release over four weeks, aiding in cervical cancer treatment and preventing its recurrence [137]. Given the unique nature of the cervix, these fibrous membranes typically possess antibacterial effects [138] and promote wound healing [139] alongside their therapeutic roles in treating cervical cancer.

**Figure 7:**
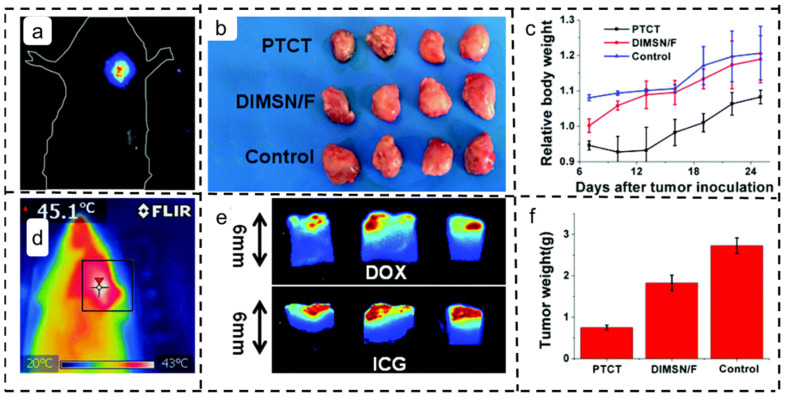
Application of chitosan/poly(vinyl alcohol) (CS/PVA) drug-loaded nanofiber scaffolds in the treatment of cervical cancer [140]. Copyright 2019, reproduced with permission from RSC. (**a**) Fluorescent (ICG) images of DIMSN/Fs subcutaneously implanted in the left axilla of mice. (**b**) Macroscopic tumor observation. (**c**) Relative tumor volume in each group. (**d**) Infrared thermal images of DIMSN/F mice after 808 nm laser irradiation. (**e**) Tumor weight. (**f**) Fluorescent images of tumor transects removed from the three mice receiving the first PTCT scan.

Combining chemotherapy with phototherapy holds significant promise in cervical cancer treatment. In their study, illustrated in Figure 7, Wang et al. developed MSNs carrying both DOX and indocyanine green (ICG) (named DIMSNs). These nanoparticles were then incorporated with CS/PVA, leading to the creation of multifunctional composite nanofibers (DIMSN/F) through electrospinning. These implantable nanofibers exhibited optimal drug accumulation and specific drug release within the vaginal region. When subjected to simulated vaginal secretion erosion, DIMSN/F displayed site-specific drug release, achieving a remarkable tumor inhibition rate of 72.5% for primary cervical/vaginal cancer. This outcome underscores its considerable potential in cervical cancer treatment [140].

### 4.4. Colon Cancer

**Figure 8:**
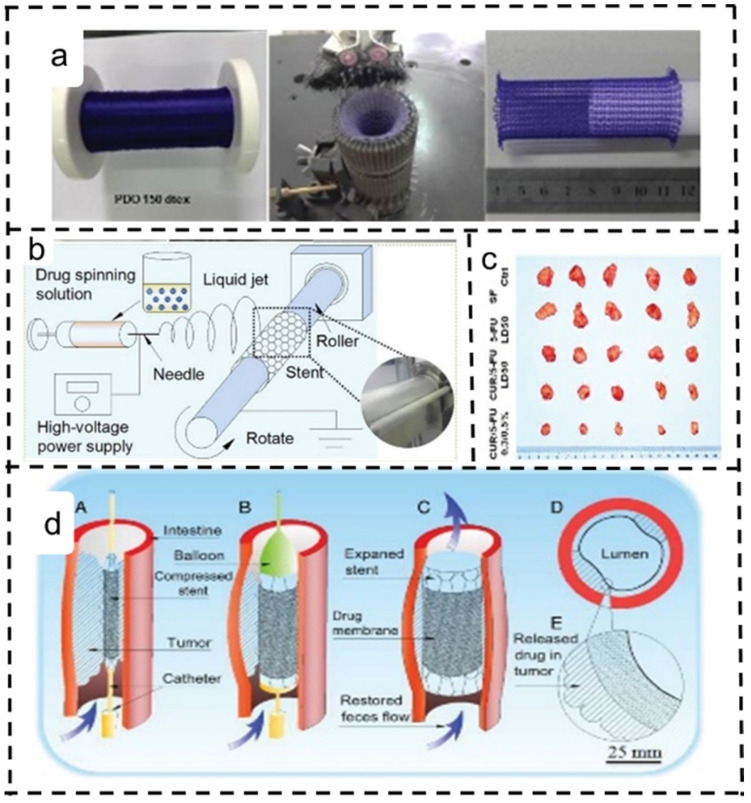
Application of expanded electrospun nanofiber scaffolds for colon cancer treatment [141]. Copyright 2018, reproduced with permission from WILEY. (**a**) Tubular stent diagram. (**b**) Schematic representation of a modified electrospinning machine. (**c**) Macroscopic images of exposed tumors. (**d**) Mechanism and process of drug-loaded stent placement and its role in colorectal cancer treatment. A, deflated balloon catheter and compressed drug-loaded stent are inserted into the stenosis site; B, inflated balloon expands the stent and compresses the tumor to restore the intestinal lumen; C, stent-widened intestine; D and E, the drugs are released from the coating membrane and the tumor is treated locally and effectively.

Colorectal cancer (CRC) stands among the most severe cancers, posing significant morbidity and mortality risks. Stent implantation, combined with radiotherapy and chemotherapy, has proven to cause effective relief in clinical practice. However, conventional metallic stents primarily address mechanical expansion needs, leading to temporary treatment. Postoperative complications, such as inward tumor growth and stent displacement due to stent stenosis, persist as unresolved issues. To address this, Xie et al. developed a fibroin-based nanofiber membrane for CRC treatment. The nanofiber membrane, loaded with a CUR/5-FU dual-drug system, exhibited enhanced antitumor effects. In practical application, a deflated balloon catheter and a compressed drug-carrying stent are inserted into a narrowed area. An inflatable balloon stent compresses the tumor, restoring the intestinal cavity and locally treating the tumor effectively (Figure 8) [141].

Another challenge in colon cancer treatment involves designing a fibrous membrane to deliver targeted drugs specifically into the intestine rather than into the stomach. This is typically achieved using a pH-sensitive substance to encapsulate and direct the drug to the precise location [65]. Rostami et al. prepared ultrathin, beadless, food-grade nanofibers using high concentration of CS, gel, and resveratrol. The presence of the gel significantly improved the electrospinning efficiency. The antioxidant activity of resveratrol-loaded nanofibers surpassed that of free resveratrol. In the gel-containing nanofibers, the release of resveratrol was hindered under acidic conditions, enabling an oral delivery mode to mitigate discomfort during implantation. Nearly half of the encapsulated resveratrol is delivered to the intestinal region [142].

### 4.5. Lung Cancer

Owing to the challenging location of lung cancer, achieving precise oral administration is unattainable, and implantable administration poses considerable pain. Hence, maximizing the drug release duration and minimizing the dosing frequency become pivotal. Mellatyar et al. investigated implantable nanofibers that enhanced the anticancer effect by inhibiting HSP90 expression and telomerase activity. PCL/PEG nanofibers loaded with 17-dimethylaminoethylamino-17-demethoxy geldanamycin (17-DMAG) exhibited superior efficacy in killing A549 lung cancer cells compared to free 17-DMAG. This was achieved by regulating heat shock protein 90 (HSP90) expression and inhibiting telomerase activity, presenting implantable 17-DMAG nanofiber scaffolds as promising tools to effectively eliminate residual lung cancer cells and prevent local tumor recurrence [143].

Samadzadeh et al. formulated nanofibers containing metformin hydro-chloride (MET) and elucidated its mechanism of action. MET primarily activates AMP-activated protein kinase (AMPK), leading to the inhibition of the mammalian target of the rapamycin (mTOR) pathway, thereby reducing protein synthesis and cancer proliferation. These fibers exhibited rapid drug release within the first day, continuing for over two weeks. Within 48 h, the MET-loaded nanofibers demonstrated significant cytotoxicity against A549 lung cancer cells. Moreover, these nanofibers effectively raised the intracellular ROS levels, inducing apoptosis in cancer cells. Compared to free MET, nanofibers with MET induced notable changes in the expression levels of Bax, Bcl-2, caspase-3, and -9 [144].

In a study by Li et al., the interaction between human lung epithelial A549 cells and electrospun nanofibers composed of PVA and type I collagen was examined by adjusting the diameter of the electrospun nanofibers. The A549 cells adhered to the nanofiber mats via a filopodia attachment and in an extended form, inducing the epithelial–mesenchymal transformation. This research into the tumor microenvironment and cancer helped elucidate the mechanism underlying lung cancer [145].

**Figure 9:**
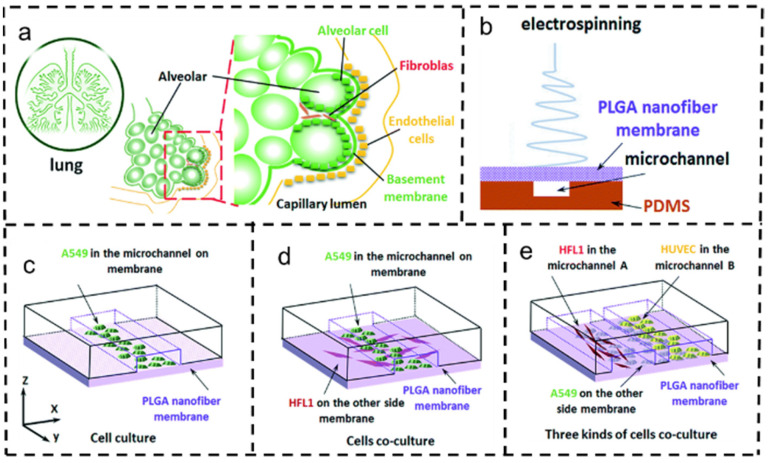
Application of electrospun fibers in exploring lung cancer mechanisms and simulating the tumor microenvironment [146]. Copyright 2018, reproduced with permission from RSC. (**a**) Schematic of alveolar structure. (**b**) Schematic of a microfluidic chip with PLGA nanofiber film as a substrate prepared by the electrospinning method. (**c**) Schematic representation of A549 cell culture on a chip. (**d**) A549 cells co-cultured with HFL1 cells. (**e**) A549 cells co-cultured with HFL1 cells and HUVECs.

Yang et al. devised a lung chip using a PLGA electrospun nanofiber membrane as a substrate, creating a cell scaffold that emulates an alveolar microenvironment. The PLGA nanofiber membrane, approximately 3 μm thick, exhibited excellent porosity, molecular permeability, and biocompatibility. Using electrospinning technology, they investigated the potential causes of drug resistance in A549 cells co-cultured with HFL1 cells. They discovered that IGF-1 secreted by HFL1 cells sustained tumor cells by activating the PI3K/Akt signaling pathway post-drug inhibition of the EGFR-related signaling pathway. This led to the reduced sensitivity of tumor cells to chemotherapy drugs (Figure 9). This study, which simulated alveolar biochemical factors in the tumor microenvironment in vitro, particularly in the respiratory membrane, holds promise for significant contributions to the local treatment of lung tumors [146].

Numerous studies have highlighted the efficacy of electrospinning technology in simulating the tumor microenvironment, studying and inhibiting tumor pathogenesis, analyzing drug resistance mechanisms, and more [133,134]. Electrospinning-assisted cancer therapy represents a novel and promising therapeutic approach.

### 4.6. Brain Cancer

The brain, the most intricate organ governing human behavior, poses significant challenges for treating tumors. Precision drug delivery using electrospun fibers emerges as a promising strategy for controlled drug loading. Glioblastoma (GBM), a prevalent malignant central nervous system tumor, notoriously resists treatment with a high recurrence rate. Temozolomide (TMZ), an alkylating agent capable of crossing the blood–brain barrier, holds promise for GBM treatment. M. Irani et al. prepared PCL-Diol-b-PU/Au composite nanofibers via electrospinning, observing the continuous delivery of TMZ in the nanofibers. An evaluation of the nanofibers’ cytotoxicity on U-87 human glioblastoma cells revealed enhanced cytotoxic effects due to the gold coating on the nanofibers’ surfaces [147].

Postoperative care for surgically resected glioblastomas typically necessitates intensive treatment, often involving implantable fibrous membranes. Molina-Peñaé et al. electrospun chitosan-mixed nanoparticles to create nanofiber scaffolds for entrapping GBM cells. These scaffolds encapsulated stromal cell-derived factor-1α (SDF-1α), sustaining the biological activity of cancer cells by inducing their migration. The scaffold facilitated the sustained release of SDF-1α for at least 5 weeks, with the nanofiber structure providing effective anchoring. This approach holds the potential for treating unresectable GBM tumors [148].

Combining photohyperthermia with chemotherapy presents numerous advantages in treating brain cancer, particularly in delicate operations. Yang et al. developed chitosan composite nanofibers incorporating self-made photosensitized CuSe nanoparticles via a green electrospinning method. After the craniotomy, these nanofibers were immediately detached upon resection, offering high accuracy (>90%) and rapid hemostasis (<8 s). Light-induced photothermal and photodynamic therapies demonstrated highly efficient bactericidal effects and effectively induced apoptosis in residual tumor cells, inhibiting tumor recurrence [149].

### 4.7. Oral Cancer

Oral cancers encompass various histological types, including adenocarcinoma, lymphoma, melanoma, and teratoma. Squamous cell cancers, accounting for around 90% of oral cancers, typically originate in the cheeks, bottom of the mouth, gums, lips, roof of the mouth, and tongue. Nam et al. employed PVA and Soluplus (SP) as raw materials to fabricate Angelica gigas Nakai (AGN)/PVA/SP nanomaterials via electrospinning, achieving an encapsulation rate of 80%. Both PVA and SP act as hydrophilic polymers and surfactants for nanofiber formulation through electrospinning processes. The developed electrospun nanofiber preparations loaded with AGN were administered multiple times into the mouth, demonstrating negligible toxicity to various organs and tissues in mice. The fiber membrane adopts an explosive release mode, ensuring drug release within the oral cavity [68].

Will et al. achieved the local release of diclofenac using electrostatically spun nanofibers made from poly(d,l-lactide-co-glycolide) polymer. Controlled experiments revealed that the implantation of these electrospun scaffolds reduced tumor recurrence and significantly improved survival over a 7-week study period post-tumor resection. This approach holds substantial potential for directly inhibiting tumor growth and curbing inflammation-mediated tumor progression [150].

**Figure 10:**
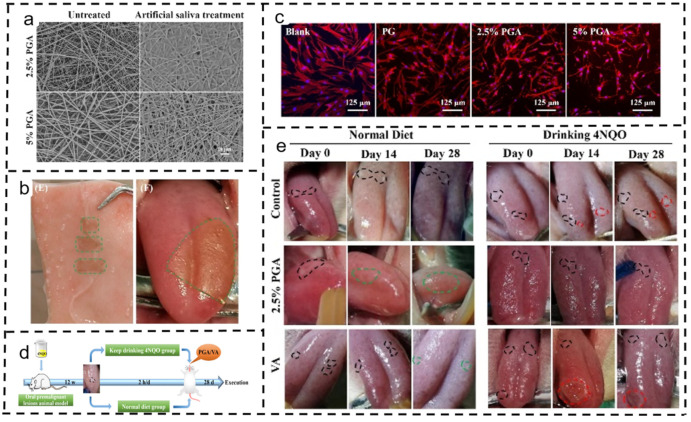
Application of polycaprolactone/gelatin (PCL/GT) nanofibers in oral cancer [151]. Copyright 2022, reproduced with permission from Elsevier. (**a**) Changes in fiber morphology of PGA electrospun membranes after soaking in artificial saliva. (**b**) Images of 2.5% PGA electrospun membrane adhesion to pig skin and rat tongue mucosa. (**c**) Fluorescence images of electrospun membranes with different ASX contents and hGFs co-cultured for 24 h. (**d**) Schematic representation of animal experiments. (**e**) Photographic representation of the PGA electrospun membrane and VA ointment treated with OPL on days 0, 14, and 28 (green dotted line indicates a good treatment effect; red dotted line indicates deterioration in treatment response; black dotted line indicates no change).

Zhang et al. pioneered an astaxanthin-based polycaprolactone/gelatin (PCL/GT) nanofiber adhesive patch (PGA) engineered with a saliva-insoluble PCL backing to prevent drug loss post-oral administration (Figure 10). This patch was designed to achieve an optimal release rate of astaxanthin, ensuring a high local drug concentration and penetration into the oral mucosa. Notably, its high porosity facilitated excellent adhesion to wet tissue and allowed good air permeability. The bioactive adhesive tablets significantly fostered the recovery of oral premalignant lesions (OPLs) by suppressing the expressions of Ki67 and cyclooxygenase-2 (COX-2). Remarkably, these tablets exhibited efficacy comparable to clinical retinoic cream preparations but without the side effects observed in the latter, such as hair loss and mouth ulcers [151].

### 4.8. Other Cancers

**Figure 11:**
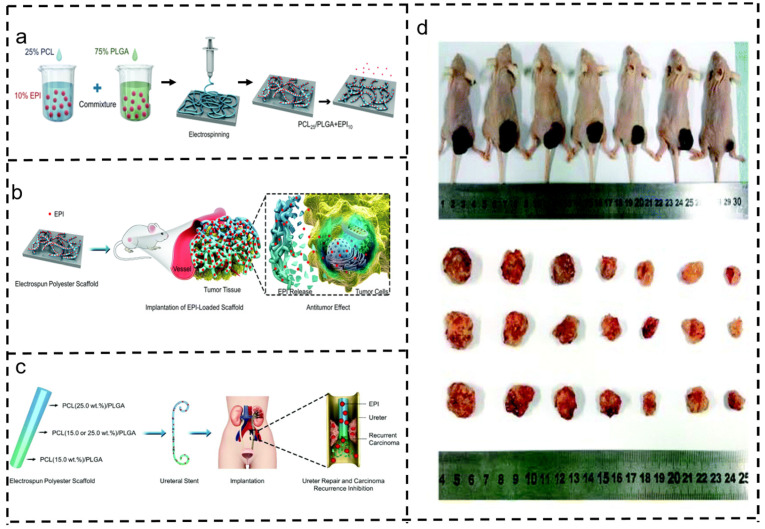
Application of fibrous membrane scaffolds in urothelial carcinoma [152]. Copyright 2019, reproduced with permission from RSC. (**a**) Stent preparation. (**b**) In vivo antitumor effect of electrospun polyester stent loaded with EPI implanted on the tumor surface. (**c**) Application diagram of electrospun polyester stent in preventing recurrence of upper urinary tract tumors and repairing the ureter in vivo. (**d**) Images of mice carrying T24 tumors after treatment and tumor resection. From left to right: control, PCL15/PLGA, PCL25/PLGA, PCL15/PLGA + EPI5, PCL15/PLGA + EPI10, PCL25/PLGA + EPI5, and PCL25/PLGA + EPI10 groups.

The challenge in treating urothelial carcinoma lies in the restricted space of the ureter, making retention difficult. For low-risk upper tract urothelial carcinoma (UTUC), renal preservation surgery is the preferred strategy. Post-surgery, preventing ureteral cancer recurrence involves supporting the ureter with a stent. J. Wang et al. investigated the controlled delivery of epirubicin (EPI) using a gradient-degraded electrospun PCL/PLGA scaffold to assess its antitumor activity against UTUC. The PCL/PLGA electrospun fiber scaffold exhibited sustained and controlled degradation, enabling regulated drug release kinetics. Specifically, as the proportion of PCL increased, the degradation and release rates slowed, aiding in post-surgery stent retention to inhibit and eliminate participating cancer cells (Figure 11) [152].

Electrospun fiber stents have been applied in urothelial carcinoma treatment. Musciacchio et al. reported that rifampicin (Rif)-supported membranes initially demonstrated rapid drug release within 24 h, followed by a slowed release while maintaining antibacterial efficacy by inhibiting bacterial proliferation and providing a bactericidal effect. The hydrophobic nature of the PCL/Rif membrane allowed for moistening by physiological liquids and cell media, ensuring cell adhesion, proliferation, and prolonged antibacterial activity against urothelial cells and smooth muscle cells, making it suitable for ureteral regeneration. This fibrous membrane stands as a potential ureteral stent for urothelial carcinoma [153].

Electrospun drug-loaded nanofibers have found diverse applications in treating various cancers. They have been employed in gastric retention administration for ascite cancer [154], continuous administration for liver cancer [155], simulating tumor microenvironments in osteosarcoma [156], detecting and locally delivering treatments in prostate cancer [157,158], and also in the prevention of pancreatic cancer [159] and gallbladder cancer [160]. Their versatility in drug delivery and targeting specific cancer types highlights their potential across a wide spectrum of oncological treatments. Unfortunately, the current application of drug-loaded electrospun nanofibers in cancer treatment is mostly limited to cell experiments and mouse experiments, with almost no relevant approved medical devices or clinical trials, as shown in Table S1.

## 5. Conclusions and Future Perspectives

Electrospinning stands as a versatile method for crafting nanoscale fibers through electrostatic forces. This technique allows for precise control over needle structures, facilitating the creation of specialized configurations like core–sheath, Janus, parallel, and eccentric structures. Moreover, adjusting the polymer composition, flow rates, and voltage settings enables the production of porous, hollow, and bead-on-string structures. These unique configurations significantly influence drug release dynamics, serving different roles at various stages of cancer treatment. However, challenges persist in scaling up electrospinning for mass production, representing a focal point for ongoing research and development efforts.

With the development of electrospinning technology, the range of raw materials that can be used to prepare nanofibers is gradually expanding. Organic compounds with good biocompatibility, various drug molecules, and even gene fragments can be used for electrospinning, and they can be combined to meet specific application requirements. Electrospun nanofibers, primarily composed of biodegradable polymers, offer versatility in cancer treatment without requiring removal, and they have been extensively studied for various applications.

Core–sheath structures, frequently utilized for gradual drug release, involve encapsulating drugs within the core layer, gradually releasing them through the hydrophobic polymer in the sheath. Fine-tuning the core–sheath thickness allows for precise control over the drug release rate, which is a prevalent strategy in cancer treatment. Recent trends include incorporating particles into electrospun fiber solutions. Magnetic electrospinning inclusion, for instance, allows for the integration of chemotherapy with magnetic, heat, or radiation therapies. This innovation significantly broadens cancer treatment options and helps combat drug resistance.

Additionally, the possibility of tailoring electrospinning to specific cancer types demonstrates its adaptability: it can be used with pH-sensitive polymers for precise delivery in colon cancer, hydrophilic polymers for rapid release in oral cancer, and floating drug-loaded fiber membranes for gastric cancer treatment. This method caters to diverse drug release and implantation needs in cancer therapy, positioning itself as a promising avenue for future applications.

## Figures and Tables

**Table 1:** Typical examples of drug-loaded electrospun fibers.

Release Mode	Fluid Composition			Spinning Condition		Loaded Drug	Released Time (h)	Application	Ref.
	Core	Middle	Sheath	Voltage (kV)	Core-to-Sheath Velocity Ratio (ml/h)				
Quick release	CA, PEG, RUT	/	CA, PEG, QUE	25	3.0; 3.0	Quercetin (QUE); Rutin (RUT)	6	Cervical cancer treatment and wound dressing	[58]
	PVP, AB, VAL	/	/	15	1.3	Amlodipine besylate; Valsartan	0.1	Hypertension treatment	[66]
	PVA, SP, AGN	/	/	25	1.0	Angelica gigas Nakai (AGN)	0.25	Oral patch, instant pad preparation for oral cancer	[67]
Sustained release	CA, MET	CA, MET	Acetone; DMAc; Ethanol	15	0–2.0; 2.0–0; 0.5	Metformin hydrochloride (MET)	23.4	Type II diabetes	[68]
	PLA	/	/	15	1.5	Theophylline	288	Treatment of bronchial asthma, asthmatic bronchitis, and obstructive emphysema	[62]
	CA, EC	/	/	15	0.5–1.0	Ketoprofen (KET)	500	Anti-inflammatory action	[69]
	EC; Berberine hydrochloride	/	Glycerol Monostearate	16	1.5; 0.5	Berberine hydrochloride	32	Dysentery, cancer, diabetes, and hyperlipidemia	[70]
	ACY	ACY, CA	CA	14	0; 0.3; 1.0/0.3; 1.0; 0.3	Acyclovir (ACY)	42	Antiviral treatment	[71]
	FA; Gliadin	CA	Acetone; Acetate Acid	15	2; 0–0.5; 0.3	Ferulic acid (FA)	40	Cancer, diabetes, and antioxidants	[72]
Biphasic release	PAN, ACY	/	ACY	18	1.5–2.0; 0.5–0	Acyclovir (ACY)	16	Various infections caused by herpes simplex virus	[9]
	CIP	/	CA	16	0.5; 0–0.1	Ciprofloxacin (CIP)	24	Biphasic drug-controlled release	[28]
	PVP	PCL, CF:TFE (3:1)	PCL, TFE	15	0–0.08; 0.4–1.2; 0.2–0.4	Dyes: key acid blue (KAB); key acid uranine (KAU)	24	Wound dressing and cancer treatment	[56]
Targeted release	DS; Ethanol	ES100; DMAc	Ethanol	5	0–0.4; 3.0–1.6; 0–1.0	Diclofenac sodium (DS)	24	Oral administration, colonic biofilm penetration	[14]
	PEO, CUR, 80% Ethanol	Ethanol	Shellac; Ethanol	6	0.6; 0.2; 0.2–0.6	Curcumin (CUR)	72	Oral colonic administration; microbead fibers serve as drug repositories	[65]
	EC, KET	EC, KET	EC, KET	12	0.2; 0.5; 2	Ketoprofen (KET)	20	Incorporated into the intestinal sol capsule, released linearly, and administered orally	[73]

## Data Availability

No new data were created or analyzed in this study. Data sharing is not applicable to this article.

## Supplementary Materials

The following supporting information can be downloaded at: https://www.mdpi.com/article/10.3390/polym16040504/s1, Table S1. The research progress of cancer treatment applications listed in the article.

## Abbreviations

Polyvinylpyrrolidone (PVP); polycaprolactone (PCL); cellulose acetate (CA); polyethylene glycol (PEG); quercetin (QUE); rutin (RUT); amlodipine besylate (AB); valsartan (VAL); poly(vinyl alcohol) (PVA); Soluplus (SP); angelica gigas Nakai (AGN); metformin hydrochloride (MET); N,N-Dimethylacetamide (DMAc); polylactic acid (PLA); ethyl cellulose (EC); ketoprofen (KET); acyclovir (ACY); polyacrylonitrile (PAN); ciprofloxacin (CIP); chloroform (CF); 2,2,2,-trifluoroethanol (TFE); key acid blue (KAB); key acid uranine (KAU); diclofenac sodium (DS); Eudragit S100 (ES100); polyethylene oxide (PEO); curcumin (CUR); Fixed-Dose Combinations (FDCs); chitosan (CS); poly(lactic-co-glycolic acid) copolymer (PLGA); resveratrol (RES); xanthohumol (XAN); γ-cyclodextrin (γ-CD); Drug Delivery Systems (DDS); tumor microenvironment (TME); 3-(diethylamino)propylamine (DEAP); paclitaxel (PTX); lidocaine hydrochloride (Lid); silybin (SB); honokiol (HNK); fatty acid amide hydrolase (FAAH); epirubicin (EPI); doxorubicin (DOX); berberine (BBR); apatinib (AP); matrix metalloproteinases-2 (MMP-2); mesoporous silica nanoparticles (MSNs); 5-Fluorouracil (5-FU); Terminalia catappa (TC); amorphous calcium carbonate (ACC); gelatin/polycaprolactone (GP); spherical silver nanoparticles (AgNPs); indocyanine green (ICG); colorectal cancer (CRC); 17-dimethylaminoethylamino-17-demethoxy geldanamycin (17-DMAG); heat shock protein 90 (HSP90); AMP-activated protein kinase (AMPK); mammalian target of the rapamycin (mTOR); glioblastoma (GBM); temozolomide (TMZ); stromal cell-derived factor-1α (SDF-1α); gelatin (GT); oral premalignant lesions (OPL); cyclooxygenase-2 (COX-2); upper tract urothelial carcinoma (UTUC); rifampicin (Rif); poly(glycolide-ε-caprolactone) (PGCL); N-carboxymethyl chitosan (N-CMCS); prodigiosin (PG); sodium alginate (ALG); capsaicin powder (Cap); graphene oxide (GO); docetaxel(DTX); cetyltrimethylammonium bromide (CTAB); Tetraethoxysilane (TEOS); N-carboxymethyl chitosan (CMC); polycaprolactone diol (PCL-Diol); sodium alginate (SA); andrographolide (AG); 2,2-diphenyl-1-picrylhydrazyl (DPPH); thyme extract (TE); acid ferulic acid (FA); diclofenac sodium salt (DSS); polydioxanone (PDO); hexamethylene diisocyanate (HDI); stromal cell-derived factor-1α (SDF-1α); poly(d,l-lactide-co-glycolide) (PDLGA); poly(L-lactic acid) (PLLA); rectorite (REC); Cabazitaxel (CTX); gallic acid (GA).

## References

[1] Fawal G.E., Abu-Serie M.M., El-Gendi H., El-Fakharany E.M. (2022). Fabrication, characterization and in vitro evaluation of disulfiram-loaded cellulose acetate/poly(ethylene oxide) nanofiber scaffold for breast and colon cancer cell lines treatment. Int. J. Biol. Macromol..

[2] Zhang Z., Yan H., Li S., Liu Y., Ran P., Chen W., Li X. (2021). Janus rod-like micromotors to promote the tumor accumulation and cell internalization of therapeutic agents. Chem. Eng. J..

[3] Yu D.-G., Huang C. (2023). Electrospun Biomolecule-Based Drug Delivery Systems. Biomolecules.

[4] Yu D.-G., Xu L. (2023). Impact Evaluations of Articles in Current Drug Delivery Based on Web of Science. Curr. Drug Deliv..

[5] Yu D.G., Zhou J. (2023). How Can Electrospinning Further Service Well for Pharmaceutical Researches?. J. Pharm. Sci..

[6] Kachwal V., Tan J. (2022). Stimuli-responsive electrospun fluorescent fibers augmented with aggregation-induced emission (AIE) for smart applications. Adv. Sci..

[7] Rezaei F., Nikiforov A., Morent R., Geyter N.D. (2018). Plasma modification of poly lactic acid solutions to generate high quality electrospun PLA nanofibers. Sci. Rep..

[8] Duan H., Chen H., Qi C., Lv F., Wang J., Liu Y., Liu Z., Liu Y. (2024). A novel electrospun nanofiber system with PEGylated paclitaxel nanocrystals enhancing the transmucus permeability and in situ retention for an efficient cervicovaginal cancer therapy. Int. J. Pharm..

[9] Lv H., Guo S., Zhang G., He W., Wu Y., Yu D. (2021). Electrospun structural hybrids of acyclovir-polyacrylonitrile at acyclovir for modifying drug release. Polymers.

[10] Khatami F., Baharian A., Akbari-Birgani S., Nikfarjam N. (2023). Tubular scaffold made by gelatin/polylactic acid nanofibers for breast ductal carcinoma in situ tumor modeling. J. Drug Deliv. Sci. Technol..

[11] Zhang C., Li Y., Wang P., Zhang A., Feng F., Zhang H. (2019). Electrospinning of bilayer emulsions: The role of gum Arabic as a coating layer in the gelatin-stabilized emulsions. Food Hydrocoll..

[12] Yan E., Jiang J., Yang X., Fan L., Wang Y., An Q., Zhang Z., Lu B., Wang D., Zhang D. (2020). pH-sensitive core-shell electrospun nanofibers based on polyvinyl alcohol/polycaprolactone as a potential drug delivery system for the chemotherapy against cervical cancer. J. Drug Deliv. Sci. Technol..

[13] Qian C.H., Liu Y.B., Chen S., Zhang C.Y., Chen X.H., Liu Y.H., Liu P. (2023). Electrospun core-sheath PCL nanofibers loaded with nHA and simvastatin and their potential bone regeneration applications. Front. Bioeng. Biotechnol..

[14] Yang C., Yu D., Pan X., Liu X., Wang S., Bligh W.A., Williams G.R. (2016). Electrospun pH-sensitive core–shell polymer nanocomposites fabricated using a tri-axial process. Acta Biomater..

[15] Brako F., Luo C., Matharu R.K., Ciric L., Harker A., Edirisinghe M., Craig D.Q.M. (2020). A Portable device for the generation of drug-loaded three-compartmental fibers containing metronidazole and iodine for topical application. Pharmaceutics.

[16] Zhang X., Chi C., Chen J., Zhang X., Gong M., Wang X., Yan J., Shi R., Zhang L., Xue J. (2021). Electrospun quad-axial nanofibers for controlled and sustained drug delivery. Mater. Des..

[17] Zheng X., Kang S., Wang K., Yang Y., Yu D., Wan F., Williams G.R., Bligh S.A. (2021). Combination of structure-performance and shape-performance relationships for better biphasic release in electrospun Janus fibers. Int. J. Pharm..

[18] Chang S., Wang M., Zhang F., Liu Y., Liu X., Yu D., Shen H. (2020). Sheath-separate-core nanocomposites fabricated using a tri-fluid electrospinning. Mater. Des..

[19] Lv J., Gu W., Cui X., Dai S., Zhang B., Ji G. (2019). Nanofiber network with adjustable nanostructure controlled by PVP content for an excellent microwave absorption. Sci. Rep..

[20] Ignatova M., Anastasova I., Manolova N., Rashkov I., Markova N., Kukeva R., Stoyanova R., Georgieva A., Toshkova R. (2022). Bio-based electrospun fibers from chitosan Schiff base and polylactide and their Cu^2+^ and Fe^3+^ complexes: Preparation and antibacterial and anticancer activities. Polymers.

[21] Contardi M., Kossyvaki D., Picone P., Summa M., Guo X., Heredia-Guerrero J.A., Giacomazza D., Carzino R., Goldoni L., Scoponi G. (2021). Electrospun polyvinylpyrrolidone (PVP) hydrogels containing hydroxycinnamic acid derivatives as potential wound dressings. Chem. Eng. J..

[22] Lian H., Meng Z. (2017). Melt electrospinning vs. solution electrospinning: A comparative study of drug-loaded poly (ε-caprolactone) fibres. Mater. Sci. Eng. C.

[23] Liu Y., Wang Q., Lu Y., Deng H., Zhou X. (2020). Synergistic enhancement of cytotoxicity against cancer cells by incorporation of rectorite into the paclitaxel immobilized cellulose acetate nanofibers. Int. J. Biol. Macromol..

[24] Wu S., Li J., Mai J., Chang M. (2018). Three-dimensional electrohydrodynamic printing and spinning of flexible composite structures for oral multidrug forms. ACS Appl. Mater. Interfaces.

[25] Wang Y., Yu D.-G., Liu Y., Liu Y.-N. (2022). Progress of Electrospun Nanofibrous Carriers for Modifications to Drug Release Profiles. J. Funct. Biomater..

[26] Yu D.-G., Zhao P. (2022). The Key Elements for Biomolecules to Biomaterials and to Bioapplications. Biomolecules.

[27] Lian H., Meng Z. (2017). Melt electrospinning of daunorubicin hydrochloride-loaded poly (ε-caprolactone) fibrous membrane for tumor therapy. Bioact. Mater..

[28] Zhou K., Wang M., Zhou Y., Sun M., Xie Y., Yu D. (2022). Comparisons of antibacterial performances between electrospun polymer@drug nanohybrids with drug-polymer nanocomposites. Adv. Compos. Hybrid Mater..

[29] Prabu G.T.V., Dhurai B. (2020). A novel profiled multi-pin electrospinning system for nanofiber production and encapsulation of nanoparticles into nanofibers. Sci. Rep..

[30] Zhou J., Wang L., Gong W., Wang B., Yu D.-G., Zhu Y. (2023). Integrating Chinese Herbs and Western Medicine for New Wound Dressings through Handheld Electrospinning. Biomedicines.

[31] Xi H., Zhao H. (2019). Silk fibroin coaxial bead-on-string fiber materials and their drug release behaviors in different pH. J. Mater. Sci..

[32] Li T., Ding X., Tian L., Ramakrishna S. (2017). Engineering BSA-dextran particles encapsulated bead-on-string nanofiber scaffold for tissue. engineering applications. J. Mater. Sci..

[33] Li T., Liu L., Wang L., Ding X. (2019). Solid drug particles encapsulated bead-on-string nanofibers: The control of bead number and its corresponding release profile. J. Biomater. Sci. Polym. Ed..

[34] Ma L., Shi X., Zhang X., Li L. (2019). Electrospinning of polycaprolacton/chitosan core-shell nanofibers by a stable emulsion system. Colloid Surf. A.

[35] Wang L., Ma D., Xu C., Gan X., Ge P., Zhu L., Wang X., Lv Y. (2023). Preparation of flexible hollow TiO_2_ fibrous membranes for thermal-insulation applications by coaxial electrospinning. Ceram. Int..

[36] Najafi S.J., Gharehaghaji A.A., Etrati S.M. (2016). Fabrication and characterization of elastic hollow nanofibrous PU yarn. Mater. Des..

[37] Zhao S., Sun J., Qin Z., Li Y., Yu H., Wang G., Gu X., Pan K. (2022). Janus-structural AIE nanofiber with white light emission and stimuli-response. Small.

[38] Zhou J., Dai Y., Fu J., Yan C., Yu D.-G., Yi T. (2023). Dual-Step Controlled Release of Berberine Hydrochloride from the Trans-Scale Hybrids of Nanofibers and Microparticles. Biomolecules.

[39] Chen S., Zhou J., Fang B., Ying Y., Yu D.G., He H. (2023). Three EHDA Processes from A Detachable Spinneret for Fabricating Drug Fast Dissolution Composites. Macromol. Mater. Eng..

[40] Fang D., Qin Z., Zheng L., Yew P.Y.M., Jiang X., Kai D., Song F., Zhao J. (2023). Ros-responsive nanocomposite scaffolds for sustained releasing puerarin toachieve chondroprotection in OA rats. Mater. Des..

[41] Shen S., Zhu L., Liu J., Ali A., Zaman A., Ahmad Z., Chen X., Chang M. (2020). Novel core-shell fiber delivery system for synergistic treatment of cervical cancer. J. Drug Deliv. Sci. Technol..

[42] Zhao L., Orlu M., Williams G.R. (2021). Electrospun fixed dose combination fibers for the treatment of cardiovascular disease. Int. J. Pharm..

[43] Cao X., Chen W., Zhao P., Yang Y., Yu D.-G. (2022). Electrospun Porous Nanofibers: Pore-Forming Mechanisms and Applications for Photocatalytic Degradation of Organic Pollutants in Wastewater. Polymers.

[44] Moghe A.K., Gupta B.S. (2008). C-axial electrospinning for nanofiber structures: Preparation and applications. Polym. Rev..

[45] Yu D., Li J., Williams G.R., Zhao M. (2018). Electrospun amorphous solid dispersions of poorly water-soluble drugs: A review. J. Control. Release.

[46] Wang H., Lu Y., Yang H., Yu D.-G., Lu X. (2023). The Influence of the Ultrasonic Treatment of Working Fluids on Electrospun Amorphous Solid Dispersions. Front. Mol. Biosci..

[47] Zheng Q., Xi Y., Weng Y. (2024). Functional electrospun nanofibers: Fabrication, properties, and applications in wound-healing Process. RSC Adv..

[48] Yu D.G., Zhou J. (2024). Electrospun multi-chamber nanostructures for sustainable biobased chemical nanofibers. Next Mater..

[49] Li J., Du Q., Wan J., Yu D.G., Tan F., Yang X. (2024). Improved synergistic anticancer action of quercetin and tamoxifen citrate supported by an electrospun complex nanostructure. Mater. Des..

[50] Alba-Perez A., Jayawarna V., Childs P.G., Dalby M.J., Salmeron-Sanchez M. (2020). Plasma polymerised nanoscale coatings of controlled thickness for efficient solid-phase presentation of growth factors. Mater. Sci. Eng. C.

[51] Nagiah N., Murdock C.J., Bhattacharjee M., Nair L., Laurencin C.T. (2020). Development of tripolymeric triaxial electrospun fibrous matrices for dual-drug delivery applications. Sci. Rep..

[52] Jouybari M.H., Hosseini S., Mahboobnia K., Boloursazd L.A., Moradie M., Irani M. (2019). Simultaneous controlled release of 5-FU, DOX and PTX from chitosan/PLA/5-FU/g-C3N--DOX/g-C3N4-PTX triaxial nanofibers for breast cancer treatment in vitro. Colloids Surf. B Biointerfaces.

[53] Chen X., Liu Y., Liu P. (2024). Electrospun Core–Sheath Nanofibers with a Cellulose Acetate Coating for the Synergistic Release of Zinc Ion and Drugs. Mol. Pharm..

[54] Wang Y., Liu L., Zhu Y., Wang L., Yu D.-G., Liu L.-y. (2023). Tri-Layer Core–Shell Fibers from Coaxial Electrospinning for a Modified Release of Metronidazole. Pharmaceutics.

[55] Liao Q., Kim E.J., Tang Y., Xu H., Yu D., Song W., Kim B.J. (2023). Rational Design of Hyper-Crosslinked Polymers for Biomedical Applications. J. Polym. Sci..

[56] Han D., Steckl A.J. (2013). Triaxial electrospun nanofiber membranes for controlled dual release of functional molecules. ACS Appl. Mater. Interfaces.

[57] Sun L., Zhou J., Chen Y., Yu D.G., Liu P. (2023). A Combined Electrohydrodynamic Atomization Method for Preparing Nanofiber/Microparticle Hybrid Medicines. Front. Bioeng. Biotechnol..

[58] Stoyanova N., Spasova M., Manolova N., Rashkov I., Georgieva A., Toshkova R. (2022). Quercetin- and rutin-containing electrospun cellulose acetate and polyethylene glycol fibers with antioxidant and anticancer properties. Polymers.

[59] Xu L., He H., Du Y., Zhang S., Yu D.-G., Liu P. (2023). Electrosprayed Core (Cellulose Acetate)–Shell (Polyvinylpyrrolidone) Nanoparticles for Smart Acetaminophen Delivery. Pharmaceutics.

[60] Ji Y., Zhao H., Liu H., Zhao P., Yu D.-G. (2023). Electrosprayed Stearic-Acid-Coated Ethylcellulose Microparticles for an Improved Sustained Release of Anticancer Drug. Gels.

[61] Yan X., Xu B., Xia C., Xu M., Zeng B., Zhang R., Zhu L., Zhang C. (2023). Dual drug-loaded core-shell nanofibers membranes via emulsion electrospinning and their controllable sustained release property. J. Drug. Deliv. Sci. Technol..

[62] Liu M., Hao X., Wang Y., Jiang Z., Zhang H. (2020). A biodegradable core-sheath nanofibrous 3D hierarchy prepared by emulsion electrospinning for sustained drug release. J. Mater. Sci..

[63] Yang G., Wang J., Li L., Ding S., Zhou S. (2014). Electrospun micelles/drug-loaded nanofibers for time-programmed multi-agent release. Macromol. Biosci..

[64] Yang Y., Chen W., Wang M., Shen J., Tang Z., Qin Y., Yu D. (2023). Engineered shellac beads-on-the-string fibers using triaxial electrospinning for improved colon-targeted drug delivery. Polymers.

[65] Guo H., Tan S., Gao L.J., Wang J. (2020). Sequential release of drugs form a dual-delivery system based on pH-responsive nanofibrous mats towards wound care. Mater. Chem. B.

[66] Bukhary H., Williams G.R., Orlu M. (2018). Electrospun fixed dose formulations of amlodipine besylate and valsartan. Int. J. Pharm..

[67] Nam S., Lee J., Lee S.Y., Jeong J.Y., Kang W., Cho H. (2017). Angelica gigas Nakai extract-loaded fast-dissolving nanofiber based on poly(vinyl alcohol) and Soluplus for oral cancer therapy. Int. J. Pharm..

[68] Xu H., Xu X., Li S., Song W., Yu D., Bligh S.W.A. (2021). The effect of drug heterogeneous distributions within core-sheath nanostructures on its sustained release profiles. Biomolecules.

[69] Um-i-Zahra S., Shen X.X., Li H., Zhu L. (2014). Study of sustained release drug-loaded nanofibers of cellulose acetate and ethyl cellulose polymer blends prepared by electrospinning and their in-vitro drug release profiles. J. Polym. Res..

[70] Hai T., Wan X., Yu D., Wang K., Yang Y., Liu Z. (2019). Electrospun lipid-coated medicated nanocomposites for an improved drug sustained-release profile. Mater. Des..

[71] Wang M., Hou J., Yu D., Li S., Zhu J., Chen Z. (2020). Electrospun tri-layer nanodepots for sustained release acyclovir. J. Alloys Compd..

[72] Liu X., Yang Y., Yu D., Zhu M., Zhao M., Williams G.R. (2019). Tunable zero-order drug delivery systems created by modified triaxial electrospinning. Chem. Eng. J..

[73] Yu D., Li X., Wang X., Yang J., Bligh S.W.A., Williams G.R. (2015). Nanofibers fabricated using triaxial electrospinning as zero order drug delivery systems. ACS Appl. Mater. Interfaces.

[74] Lemraski E.G., Alibeigi S., Abbasi Z. (2022). Ibuprofen@silver loaded on poly(vinyl alcohol)/chitosan co-polymer scaffold as a novel drug delivery system. Mater. Today Commun..

[75] Tawfik E.A., Craig D.Q.M., Barker S.A. (2020). Dual drug-loaded coaxial nanofibers for the treatment of corneal abrasion. Int. J. Pharm..

[76] Zhang X., Han L., Sun Q., Xia W., Zhou Q., Zhang Z., Song X. (2020). Controlled release of resveratrol and xanthohumol via coaxial electrospinning fibers. J. Biomater. Sci. Polym. Ed..

[77] Niiyama E., Uto K., Lee C.M., Sakura K., Ebara M. (2019). Hyperthermia nanofiber platform synergized by sustained release of paclitaxel to improve antitumor efficiency. Adv. Healthc. Mater..

[78] Gao S., Zhou A., Cao B., Wang J., Li F., Tang G., Jiang Z., Yang A., Xiong R., Lei J. (2021). A tunable temperature-responsive and tough platform for controlled drug delivery. New J. Chem..

[79] Khan I., Pandit J., Ahmed S., Zameer S., Ahmad S., Bano S., Ansari M.D., Solanki P., Jahan R.N. (2022). Development and evaluation of biodegradable polymeric lomustine nanofibres for the efficient tumor targeting: In vitro characterization, ex vivo permeation and degradation study. J. Drug Deliv. Sci. Technol..

[80] Li X., He Y., Hou J., Yang G., Zhou S. (2020). A time-programmed release of dual drugs from an implantable trilayer structured fiber device for synergistic treatment of breast cancer. Small.

[81] García-García P., Reyes R., Segredo-Morales E., Pérez-Herrero E., Delgado A., Évora C. (2019). PLGA-BMP-2 and PLA-17β-estradiol microspheres reinforcing a composite hydrogel for bone regeneration in osteoporosis. Pharmaceutics.

[82] Bourdon L., Attik N., Belkessam L., Chevalier C., Bousige C., Brioude A., Salles V. (2023). Direct-writing electrospun functionalized scaffolds for periodontal regeneration: In vitro studies. J. Funct. Biomater..

[83] Sun M., Liu Y., Jiao K., Jia W., Jiang K., Cheng Z., Liu G., Luo Y. (2022). A periodontal tissue regeneration strategy via biphasic release of zeolitic imidazolate framework-8 and FK506 using a uniaxial electrospun Janus nanofiber. J. Mater. Chem. B.

[84] Zhu Y., Song F., Ju Y., Huang L., Zhang L., Tang C., Yang H., Huang C. (2019). NAC-loaded electrospun scaffolding system with dual compartments for the osteogenesis of rBMSCs in vitro. Int. J. Nanomed..

[85] Ullah A., Saito Y., Ullah S., Haider M.K., Nawaz H., Duy-Nam P., Kharaghani D., Kim I.S. (2021). Bioactive Sambong oil-loaded electrospun cellulose acetate nanofibers: Preparation, characterization, and in-vitro biocompatibility. Int. J. Biol. Macromol..

[86] Ye P., Wei S., Luo C., Wang Q., Li A., Wei F. (2020). Long-term effect against methicillin-resistant staphylococcus aureus of emodin released from coaxial electrospinning nanofiber membranes with a biphasic profile. Biomolecules.

[87] Akbarzadeh M., Pezeshki-Modaress M., Zandi M. (2020). Biphasic, tough composite core/shell PCL/PVA-GEL nanofibers for biomedical application. J. Appl. Polym. Sci. A.

[88] Su Y., McCarthy A., Wong S.L., Hollins R.R., Wang G., Xie J. (2021). Simultaneous delivery of multiple antimicrobial agents by biphasic scaffolds for effective treatment of wound biofilms. Adv. Healthc. Mater..

[89] Alkaissy R., Richard M., Morris H., Snelling S., Pinchbeck H., Carr A., Mouthuy P. (2022). Manufacture of soft-hard implants from electrospun filaments embedded in 3d printed structures. Macromol. Biosci..

[90] Sahu D.K., Pradhan D., Halder J., Biswasroy P., Kar B., Ghosh G., Rath G. (2022). Design and optimization of gatifloxacin loaded polyvinyl alcohol nanofiber for the treatment of dry eye infection: In vitro and in vivo evaluation. J. Drug Deliv. Sci. Technol..

[91] Khan A.R., Nadeem M., Bhutto M.A., Yu F., Xie X., El-Hamshary H., El-Faham A., Ibrahim U.A., Mo X. (2019). Physico-chemical and biological evaluation of PLCL/SF nanofibers loaded with oregano essential oil. Pharmaceuticsal.

[92] Bikiaris N.D., Koumentakou I., Michailidou G., Kostoglou M., Vlachou M., Barmpalexis P., Karavas E., Papageorgiou G.Z. (2022). Investigation of molecular weight, polymer concentration and process parameters factors on the sustained release of the anti-multiple-sclerosis agent teriflunomide from poly(ε-caprolactone) electrospun nanofibrous matrices. Pharmaceuticals.

[93] Boncu T.E., Ozdemir N. (2022). Electrospinning of ampicillin trihydrate loaded electrospun PLA nanofibers I: Effect of polymer concentration and PCL addition on its morphology, drug delivery and mechanical properties. Int. J. Polym. Mater. Polym. Biomater..

[94] Kabay G., Demirci C., Can G.K., Meydan A.E., Daşan B.G., Mutlu M. (2018). A comparative study of single-needle and coaxial electrospun amyloid-like protein nanofibers to investigate hydrophilic drug release behavior. Int. J. Biol. Macromol..

[95] Schifino G., Gasparini C., Drudi S., Giannelli M., Sotgiu G., Posati T., Zamboni R., Treossi E., Maccaferri E., Giorgini L. (2022). Keratin/Polylactic acid/graphene oxide composite nanofibers for drug delivery. Int. J. Pharm..

[96] Zhou J., Yi T., Zhang Z., Yu D.G., Liu P., Wang L., Zhu Y. (2023). Electrospun Janus Core (Ethyl Cellulose//Polyethylene Oxide) @ Shell (Hydroxypropyl Methyl Cellulose Acetate Succinate) Hybrids for An Enhanced Colon-Targeted Prolonged Drug Absorbance. Adv. Compos. Hybrid Mater..

[97] Altinbasak I., Kocak S., Colby A.H., Alp Y., Sanyal R., Grinstaff M.W., Sanyal A. (2023). pH-Responsive nanofiber buttresses as local drug delivery devices. Biomater. Sci..

[98] Zhang Y., Lu Y., Li Y., Xu Y., Song W. (2024). Poly(Glutamic Acid)-Engineered Nanoplatforms for Enhanced Cancer Phototherapy. Curr. Drug Deliv..

[99] Yu H.S., Lee E.S. (2020). Honeycomb-like pH-responsive γ-cyclodextrin electrospun particles for highly efficient tumor therapy. Carbohydr. Polym..

[100] Graham-Gurysh E.G., Murthy A.B., Moore K.M., Hingtgena S.D., Bacheldera E.M., Ainslie K.M. (2020). Synergistic drug combinations for a precision medicine approach to interstitial glioblastoma therapy. J. Control. Release.

[101] Anders C.K., Carey L.A. (2009). Biology, metastatic patterns, and treatment of patients with triple-negative breast cancer. Clin. Breast Cancer.

[102] Al-Attar T., Madihally S.V. (2018). Influence of controlled release of resveratrol from electrospun fibers in combination with siRNA on leukemia cells. Eur. J. Pharm. Sci..

[103] Cheng H., Yang X., Che X., Yang M., Zhai G. (2018). Biomedical application and controlled drug release of electrospun fibrous materials. Mater. Sci. Eng. C.

[104] Li X., Yu N., Li J., Bai J., Ding D., Tang Q., Xu H. (2020). Novel “carrier-free” nanofiber codelivery systems with the synergistic antitumor effect of paclitaxel and tetrandrine through the enhancement of mitochondrial apoptosis. ACS Appl. Mater. Interfaces.

[105] Lübtow M.M., Hahn L., Haider M.S., Luxenhofer R. (2017). Title of the article. J. Am. Chem. Soc..

[106] Huo M., Wang H., Zhang Y., Cai H., Zhang P., Li L., Zhou J., Yin T. (2020). Co-delivery of silybin and paclitaxel by dextran-based nanoparticles for effective anti-tumor treatment through chemotherapy sensitization and microenvironment modulation. J. Control. Release.

[107] Wang H., Wang S., Wang R., Wang X., Jiang K., Xie C., Zhan C., Wang H., Lu W. (2019). Co-delivery of paclitaxel and melittin by glycopeptide-modified lipodisks for synergistic anti-glioma therapy. Nanoscale.

[108] Wang Z., Li X., Wang D., Zou Y., Qu X., He C., Deng Y., Jin Y., Zhou Y., Zhou Y. (2017). Concurrently suppressing multidrug resistance and metastasis of breast cancer by co-delivery of paclitaxel and honokiol with pH-sensitive polymeric micelles. Acta Biomater..

[109] Slivicki R.A., Xu Z., Mali S.S., Hohmann A.G. (2019). Brain permeant and impermeant inhibitors of fatty-acid amide hydrolase suppress the development and maintenance of paclitaxel-induced neuropathic pain without producing tolerance or physical dependence in vivo and synergize with paclitaxel to reduce tumor cell line viability in vitro. Pharmacol. Res..

[110] Lin F., Chen P., Wei K., Huang C., Wang C., Yang H. (2017). Rapid in situ MRI traceable gel-forming dual-drug delivery for synergistic therapy of brain tumor. Theranostics.

[111] Mozaffari S., Seyedabadi S., Alemzadeh E. (2022). Anticancer efficiency of doxorubicin and berberine-loaded PCL nanofibers in preventing local breast cancer recurrence. J. Drug Deliv. Sci. Technol..

[112] Kumar R., Uppal S., Mansi K., Das J., Pandey S.K., Kaur K., Mehta S.K. (2022). Ultrasonication induced synthesis of TPGS stabilized clove oil nanoemulsions and their synergistic effect against breast cancer cells and harmful bacteria. J. Mol. Liq..

[113] Mehnath S., Chitra K., Karthikeyan K., Jeyaraj M. (2020). Localized delivery of active targeting micelles from nanofibers patch for effective breast cancer therapy. Int. J. Pharm..

[114] Fawal G.E., Abu-Serie M.M., Mo X., Wang H. (2021). Diethyldithiocarbamate/silk fibroin/polyethylene oxide nanofibrous for cancer therapy: Fabrication, characterization and in vitro evaluation. Int. J. Biol. Macromol..

[115] Ignatova M., Manolova N., Rashkov I., Markova N., Kukeva R., Stoyanova R., Georgieva A., Oshkova R.T. (2021). 8-Hydroxyquinoline-5-sulfonic acid-containing poly(vinyl alcohol)/chitosan electrospun materials and their Cu^2+^ and Fe^3+^ complexes: Preparation, Antibacterial, Antifungal and Antitumor Activities. Polymers.

[116] Rabionet M., Yeste M., Puig T., Ciurana J. (2017). Electrospinning PCL scaffolds manufacture for three-dimensional breast cancer cell culture. Polymers.

[117] Jaworska J., Smolarczyk R., Musiał-Kulik M., Cicho’n T., Karpeta-Jarząbek P., Włodarczyk J., Stojko M., Janeczek H., Kordyka A., Kaczmarczyk B. (2021). Electrospun paclitaxel delivery system based on PGCL/PLGA in local therapy combined with brachytherapy. Int. J. Pharm..

[118] Abasalta M., Asefnejad A., Khorasani M.T., Saadatabadi A.R. (2022). Adsorption and sustained release of doxorubicin from N-carboxymethyl chitosan/polyvinyl alcohol/poly(ε-caprolactone) composite and core-shell nanofibers. J. Drug Deliv. Sci. Technol..

[119] Li X., Xu F., He Y., Li Y., Hou J., Yang G., Zhou S. (2020). A Hierarchical Structured Ultrafine Fiber Device for Preventing Postoperative Recurrence and Metastasis of Breast Cancer. Adv. Funct. Mater..

[120] Akpan U.M., Pellegrini M., Obayemi J.D., Ezenwafor T., Browl D., Ani C.J., Yipo D., Salifu A., Dozie-Nwachukwu S., Odusanya S. (2020). Prodigiosin-loaded electrospun nanofibers scaffold for localized treatment of triple negative breast cancer. Mater. Sci. Eng. C.

[121] Ahmady A.R., Solouk A., Saber-Samandari S., Akbari S., Ghanbari H., Brycki B.E. (2023). Capsaicin-loaded alginate nanoparticles embedded polycaprolactone-chitosan nanofibers as a controlled drug delivery nanoplatform for anticancer activity. J. Colloid Interface Sci..

[122] Yang X., Yang N., Zhang L., Zhao D., Lei H., Cheng S., Ge J., Ma X., Ni C., Liu Z. (2022). Eddy current thermal effect based on magnesium microrods for combined tumor therapy. Chem. Eng. J..

[123] Samadi S., Moradkhani B.M., Beheshti H., Irani M., Aliabadi M. (2018). Fabrication of chitosan/poly(lactic acid)/graphene oxide/TiO_2_ composite nanofibrous scaffolds for sustained delivery of doxorubicin and treatment of lung cancer. Int. J. Biol. Macromol..

[124] Sadeghi M., Falahi F., Akbari-Birgani S., Nikfarjam N. (2023). Trilayer tubular scaffold to mimic ductal carcinoma breast cancer for the study of chemo-photothermal therapy. ACS Appl. Polym. Mater..

[125] Maleki H., Doostan M., Shojaei S., Doostan M., Stamatis H., Gkantzou E., Bonkdar A., Khoshnevisan K. (2023). Nanofiber-based systems against skin cancers: Therapeutic and protective approaches. J. Drug Deliv. Sci. Technol..

[126] Abasalta M., Asefnejad A., Khorasani M.T., Saadatabadi A.R. (2021). Fabrication of carboxymethyl chitosan/poly(ε-caprolactone)/doxorubicin/nickel ferrite core-shell fibers for controlled release of doxorubicin against breast cancer. Carbohydr. Polym..

[127] Mohebian Z., Babazadeh M., Zarghami N., Mousazadeh H. (2021). Anticancer efficiency of curcumin-loaded mesoporous silica nanoparticles/nanofiber composites for potential postsurgical breast cancer treatment. J. Drug Deliv. Sci. Technol..

[128] Farboudi A., Nouri A., Shirinzad S., Sojoudi P., Davaran S., Akrami M., Irani M. (2020). Synthesis of magnetic gold coated poly (ε-caprolactonediol) based polyurethane/poly(N-isopropylacrylamide)-grafted-chitosan core-shell nanofibers for controlled release of paclitaxel and 5-FU. Int. J. Biol. Macromol..

[129] Munaweera I., Levesque-Bishop D., Shi Y., Di Pasqua A.J., Balkus K.J.J. (2014). Radiotherapeutic bandage based on electrospun polyacrylonitrile containing holmium-166 iron garnet nanoparticles for the treatment of skin cancer. ACS Appl. Mater. Interfaces.

[130] Stoyanova N., Spasova M., Manolova N., Rashkov I., Georgieva A., Toshkova R. (2020). Antioxidant and antitumor activities of novel quercetin-loaded electrospun cellulose acetate/polyethylene glycol fibrous materials. Antioxidants.

[131] Guo M., Zhou G., Liu Z., Liu J., Tang J., Xiao Y., Xu W., Liu Y., Chen C. (2018). Direct site-specific treatment of skin cancer using doxorubicin-loaded nanofibrous membranes. Sci. Bull..

[132] Samadzadeh S., Babazadeh M., Zarghami N., Pilehvar-Soltanahmadi Y., Mousazadeh H. (2021). An implantable smart hyperthermia nanofiber with switchable, controlled and sustained drug release: Possible application in prevention of cancer local recurrence. Mater. Sci. Eng. C.

[133] Muthulakshmi L., Prabakaran S., Ramalingam V., Rajulu A.V., Rajan M., Ramakrishna S., Luo H. (2022). Sodium alginate nanofibers loaded Terminalia catappa scaffold regulates intrinsic apoptosis signaling in skin melanoma cancer. Process Biochem..

[134] Xue C., Li M., Sutrisno L., Yan B., Zhao Y., Hu Y., Cai K., Zhao Y., Yu S., Luo Z. (2021). Bioresorbable scaffolds with biocatalytic chemotherapy and in situ microenvironment modulation for postoperative tissue repair. Adv. Funct. Mater..

[135] Çimen C.G., Dündar M.A., Kars M.D., Avcı A. (2022). Enhancement of PCL/PLA electrospun nanocomposite fibers comprising silver nanoparticles encapsulated with thymus vulgaris l. molecules for antibacterial and anticancer activities. ACS Biomater. Sci. Eng..

[136] Aggarwal U., Goyal A.K., Rath G. (2017). Development and characterization of the cisplatin loaded nanofibers for the treatment of cervical cancer. Mater. Sci. Eng. C.

[137] Chen Y., Liu Y., LeeL D., Qiu J.T., Lee T., Liu S. (2019). Biodegradable andrographolide-eluting nanofibrous membranes for the treatment of cervical cancer. Int. J. Nanomed..

[138] Yakub G., Ignatova M., Manolova N., Rashkov I., Toshkova R., Georgieva A., Markova N. (2018). Chitosan/ferulic acid-coated poly(ε-caprolactone) electrospun materials with antioxidant, antibacterial and antitumor properties. Int. J. Biol. Macromol..

[139] Šišková A.O., Cková M.B., Kroneková Z., Kleinová A., Nagy Š., Rydz J., Opálek A., Sláviková M., Andicsová A.E. (2021). The drug-loaded electrospun poly(ε-caprolactone) mats for therapeutic application. Nanomaterials.

[140] Wang X., Wang L., Zong S., Qiu R., Liu S. (2019). Use of multifunctional composite nanofibers for photothermal chemotherapy to treat cervical cancer in mice. Biomater. Sci..

[141] Xie X., Zheng X., Han Z., Chen Y., Zheng Z., He X., Wang Y., Kaplan D.L., Li Y., Li G. (2018). A biodegradable stent with surface functionalization of combined-therapy drugs for colorectal cancer. Adv. Healthc. Mater..

[142] Rostami M., Ghorbani M., MohammadiM M.A., Delavar M., Tabibiazar M., Ramezani S. (2019). Development of resveratrol loaded chitosan-gellan nanofiber as a novel gastrointestinal delivery system. Int. J. Biol. Macromol..

[143] Mellatyar H., Talaei S., Pilehvar-Soltanahmadi Y., Dadashpour M., Barzegar A., Akbarzadeh A., Zarghami N. (2018). 17-DMAG-loaded nanofibrous scaffold for effective growth inhibition of lung cancer cells through targeting HSP90 gene expression. Biomed. Pharmacother..

[144] Samadzadeh S., Mousazadeh H., Ghareghomi S., Dadashpour M., Babazadeh M., Zarghami N. (2021). In vitro anticancer efficacy of Metformin-loaded PLGA nanofibers towards the post-surgical therapy of lung cancer. J. Drug Deliv. Sci. Technol..

[145] Li X., Yan S., Dai J., Lu Y., Wang Y., Sun M., Gong J., Yao Y. (2018). Human lung epithelial cells A549 epithelial-mesenchymal transition induced by PVA/Collagen nanofiber. Colloids Surf. B Biointerfaces.

[146] Yang X., Li K., Zhang X., Liu C., Guo B., Wen W., Gao X. (2018). Nanofiber membrane supported lung-on-a-chip microdevice for anti-cancer drug testing. Lab. Chip.

[147] Irani M., Sadeghi G.M.M., Haririan I. (2017). The sustained delivery of temozolomide from electrospun PCL-Diol-b-PU/gold nanocomposite nanofibers to treat glioblastoma tumors. Mater. Sci. Eng. C.

[148] Molina-Peña R., Mansor M.H., Najberg M., Thomassin J., Gueza B., Alvarez-Lorenzo C., Garcion E., Jérôme C., Boury F. (2021). Nanoparticle-containing electrospun nanofibrous scaffolds for sustained release of SDF-1α. Int. J. Pharm..

[149] Yang J., Xu L., Ding Y., Liu C., Wang B., Yu Y., Hui C., Ramakrishna S., Zhang J., Long Y. (2023). NIR-II-Triggered Composite Nanofibers to Simultaneously Achieve Intracranial Hemostasis, Killing Superbug and Residual Cancer Cells in Brain Tumor Resection Surgery. Adv. Fiber Mater..

[150] Will O.M., Purcz N., Chalaris A., Heneweer C., Boretius S., Purcz L., Nikkola L., Ashammakhi N., Kalthoff H., Glüer C. (2016). Increased survival rate by local release of diclofenac in a murine model of recurrent oral carcinoma. Int. J. Nanomed..

[151] Zhang H., Ji Y., Yuan C., Sun P., Xu Q., Lin D., Han Z., Xu X., Zhou Q., Deng J. (2022). Fabrication of astaxanthin-loaded electrospun nanofiber-based mucoadhesive patches with water-insoluble backing for the treatment of oral premalignant lesions. Mater. Design.

[152] Wang J., Wang G., Shan H., Wang X., Wang C., Zhuang X., Ding J., Chen X. (2019). Gradiently degraded electrospun polyester scaffolds with cytostatic for urothelial carcinoma therapy. Biomater. Sci..

[153] Musciacchio L., Mardirossian M., Guagnini B., Raffini A., Rizzo M., Trombetta C., Liguori G., Turco G., Porrelli D. (2022). Rifampicin-loaded electrospun polycaprolactone membranes: Characterization of stability, antibacterial effects and urotheliocytes proliferation. Mater. Design.

[154] Anothra P., Pradhan D., Naik P.K., Ghosh G., Rath G. (2021). Development and characterization of 5-fluorouracil nanofibrous film for the treatment of stomach cancer. J. Drug Deliv. Sci. Technol..

[155] Tu H., Dai F., Cheng G., Yuan M., Zhou X., Wang Y., Zhang R., Zheng Y., Cheng Y., Deng H. (2021). Incorporation of Layered Rectorite into Biocompatible Core–Sheath Nanofibrous Mats for Sustained Drug Delivery. ACS Biomater. Sci. Eng..

[156] Molina E.R., Chim L.K., Salazar M.C., Mehta S.M., Menegaz B.A., Lamhamedi-Cherradi S., Satish T., Mohiuddin S., McCall D., Zaske A.M. (2019). Mechanically tunable coaxial electrospun models of YAP/TAZ mechanoresponse and IGF-1R activation in osteosarcoma. Acta Biomater..

[157] Rajendran K., Kokulnathan T., Chen S., Allen J.A., Viswanathan C., Therese H.A. (2019). Nitrogen doped carbon nanofibers loaded with hierarchical vanadium tetrasulfide for the voltammetric detection of the non-steroidal anti-prostate cancer drug nilutamide. Microchim. Acta.

[158] Jaworska J., Orchel A., Kaps A., Jaworska-Kik M., Hercog A., Stojko M., Włodarczyk J., Musiał-Kulik M., Pastusiak M., Bochenek M. (2022). Bioresorbable nonwoven patches as taxane delivery systems for prostate cancer treatment. Pharmaceutics.

[159] Zhan Q., Shen B., Fang Y., Deng X., Chen H., Jin J., Peng C., Li H. (2017). Drug-eluting scaffold inhibited in vivo pancreatic tumorigenesis by engaging murine CCR4+CD8+ T cells. Colloid Surf. B.

[160] Acevedo F., Hermosilla J., Sanhueza C., Mora-Lagos B., Fuentes I., Rubilar M., Concheiro A., Alvarez-Lorenzo C. (2018). Gallic acid loaded PEO-core/zein-shell nanofibers for chemopreventive action on gallbladder cancer cells. Eur. J. Pharm. Sci..

