# Supplementary material for: Application of Electrospun Drug-Loaded Nanofibers in Cancer Therapy
Source: Polymers (Basel). 2024 Feb 12;16(4):504. doi: 10.3390/polym16040504 (PMC10892891; doi:10.3390/polym16040504)
Supplement: Supplementary file 1 [file polymers-16-00504-s001.zip › polymers-2826705-supplementary.pdf]

Table S1. The research progress of cancer treatment applications listed in the article.

Cancer Type	Fluid Composition	Drugs	Cell Test	Mouse Test	Ref.
Breast cancer	PGCL; PLGA	PTX	√	√	[117]
	N-CMCS; PVA; PCL	DOX	√		[118]
	PEG; PLA	DOX	√		[119]
	PLGA; GT; pluronic F127	PG	√		[120]
	ALG; CS; PCL	Cap	√		[121]
	Mg; PCL	DOX	√	√	[122]
	CS; PLA; GO; TiO2	DOX	√		[123]
	Alginate dopamine; PVA; PCL	DTX	√		[124]
	CMC; polyvinyl alcohol; PCL	DOX	√		[126]
	SiO_2; PLGA	CUR	√		[127]
	PCL- Diol	PTX	√	√	[128]
Skin cancer	CA; PEG	QUE	√		[130]
	PLGA; PCL	DOX	√	√	[131]
	CTAB; TEOS; iron oxide (II, III); SiO_2	MET	√		[132]
	SA; DPPH	TC	√		[133]
	GT; GP	ACC	√	√	[134]
	PLA; PCL	AgNPs; TE	√		[135]
Cervical cancer	PCL; CS	cisplatin	√	√	[136]
	PLGA	AG		√	[137]
	PCL; CS	FA	√		[138]
	PCL	DSS	√		[139]
	CS; PVA	DOX; ICG		√	[140]
Colon cancer	SF; PDO	CUR; 5-FU	√	√	[141]
	CS; PEO; PCL	Lid; CUR	√		[65]
	CS; gellan	RES	√		[142]
Lung cancer	PCL; PEG	17-DMAG	√		[143]
	PLGA	MET	√		[144]
	PVA; collagen	/	√		[145]
	PLGA	/	√		[146]
Brain cancer	PCL-Diol; HDI	TMZ	√		[147]
	PLGA	SDF-1α	√	√	[148]
	CS; PEO	CuSe	√	√	[149]
Oral cancer	CA	MET			[68]
	PDLGA	Diclofenac	√	√	[150]
	PCL; GT	astaxanthin	√	√	[151]
Other cancers	PCL; PLGA	EPI	√	√	[152]
	PCL	Rif	√		[153]
	ES100	5-FU	√	√	[154]
	PLLA; PEO; REC	DOX	√	√	[155]
	PGCL; PLGA	CTX	√		[158]
	Polyglyconate; porcine gelatin	CCL17	√	√	[159]
	PEO; zein	GA	√		[160]
